# Fine-grained evaluation of neighborhood quality in China using street view images and big data technologies

**DOI:** 10.1038/s41598-026-53650-w

**Published:** 2026-06-01

**Authors:** Zhaowei Wu, Shanlang Lin, Rongwei Gao, Shouming Chen, Zeyu Lin

**Affiliations:** 1 School of Economics and Management, Tongji University, Shanghai, People’s Republic of China; 2 Shanghai International College of Intellectual Property, Tongji University, Siping Road 1239, Shanghai, 200092 People’s Republic of China

**Keywords:** Neighborhood quality, Street view images, Deep learning, Construction of indicators, Environmental social sciences, Geography, Geography

## Abstract

With the advent of the post-materialist and post-industrialization era, quality of place has gradually become a hotspot of urban research. But the research has mainly focused on the city or national scale, and there is a lack of exploration on the small scale within the city. In this paper, we take neighborhoods as the research scale, design a neighborhood quality assessment system that can measure both the soft and hard environments of cities by using big data technologies such as deep learning and street view images to obtain microdata at the neighborhood level, and the entropy method was used to assess the quality of 5829 major neighborhoods (sub-districts) in 232 prefecture-level cities across China. The assessment results demonstrate that: (1) In China, the construction of “soft” environment is more important for enhancing the neighborhood quality. (2) Most of the high-quality neighborhoods in China are located in its economically developed regions, indicating that the enhancement of neighborhood quality is, to some extent, dependent on a region’s economic level. (3) High-quality neighborhoods have been constructed in a relatively balanced manner in all dimensions, while low-quality neighborhoods show obvious deficiencies in the dimension of creativity. (4) Lastly, this paper includes a heterogeneity analysis on the neighborhood quality of cities in different regions, at different administrative levels, and with different industrial focuses, which reveals that even though China’s construction efforts on “hard” environments of cities are pretty much the same, significant differences exist in those on “soft environment”. This paper not only enriches the body of literature in the field of place quality but also provides significant theoretical support for urban planners and policymakers in planning and design, urban renewal, and the optimization of urbanization policies.

**Supplementary Information:**

The online version contains supplementary material available at 10.1038/s41598-026-53650-w.

## Introduction

As an inevitable outcome of social and economic development and an important engine for modernization and economic growth, urbanization is considered an irresistible trend of the development of human society^1^. By 2023, the urbanization rate in China has reached 66.2%^2^. Promoting the human-centered new urbanization has become a major goal under the urbanization policies developed by the Chinese government. Consequently, China’s urbanization has shifted from speed-oriented in the past to quality-oriented at present^3^. Under such macro background, city quality of human-scale has become one of the current concerns. Human-scale is a visible, touchable and palpable urban scale closely related with the human body, and neighborhood is an urban unit of human-scale^4^. Studies on city quality used to focus on elements of national, regional, urban and other macro scales, such as population density, land use mix, and accessibility. As cell phone signaling, point of interest (POI) data, Internet location service data and other big data spring up in recent years, some scholars have begun to conduct study on city quality at a micro level, such as neighborhood.

When it comes to study on neighborhood quality, the relevant literatures were originated from studies on comfort and quality of place. The concept of “local comfort” was proposed in the discussions about quality of life in 1950s, referring to the pleasant living conditions^5^ and natural and human environments, etc. in a place^6^, also including the pleasant facilities unique to that place which attract people to live and work there^7^, as well as specific goods and services that are attractive for living or working^8^. The concept of “quality of place” was first put forward by planning and design scholars on the basis of “quality of life” and “local comfort”, focusing on shaping physical space. For instance, Smith et al.^9^ referring to community quality as providing a physical environment that satisfies residents’ needs and desires, including such spaces as parks, sidewalks, schools, parking lots, streets. In their study on creative class, Arora et al.^10^ proposed, based on the living needs of the creative class, that quality of place means “a series of goods and services that fall under the extensive category of amenities”. Florida^11^ believed that quality of place includes a series of factors such as improved infrastructure, diverse leisure and cultural facilities, and a safe and inclusive social environment, all of which together make a city a place of residence that is attractive to talents. Andrews^12^ defined quality of place as an “aggregate measure of the factors in the external environment that contribute to quality of life”, including the sense of happiness and satisfaction, as well as factors that decide the quality of local life such as cultural facilities, crime rate, greening and crowding.

The evaluation indicators for quality of place have always been a key subject of urban study. Despite their diversity, the indicator systems available for evaluating quality of place are mainly divided into two types. The first is subjective evaluation indicators system, including people’s sense of happiness, satisfaction and belonging of DeMiglio and Williams^13^, which conducts qualitative and non-spatial measurement on the micro data of samples using visits or questionnaires, the other is objective evaluation indicator system, including infrastructure, social environment and ecology of a city. After a review of the literature, Glaeser et al.^14^ believed that the indicators for quality of place should incorporate environmental indicators with an impact on human health, basic indicators such as entertainment facilities, natural landscape and street view, as well as social indicators such as housing and accessibility, job opportunities, education opportunities, crime rate, social stability, government credibility, and citizen participation. By analyzing the relevant literature of Florida, Trip^15^ compiled 10 key indicators, including: urban diversity, recreational amenities, vibrancy and culture, technical innovation, talent, creativity, tolerance and openness, urban aesthetics, urban environment, and impact. However, some scholars have different views about the indicators for evaluating quality of place. Borén and Young^16^, for example, through a survey in Stockholm, Sweden, found that culture and outdoor facilities are not main factors for quality of place. According to Hansen and Niedomysl^17^, “soft” elements in human environment contain extensive factors, and while most literature uses a simple conceptual narrative, we know very little about residents’ specific preferences and behaviors.

Trip^15^ pointed out that assessment of quality of place shall pay attention to the issue of spatial scale: the assessment elements cannot be the same for countries, regions, cities and intra-city neighborhoods. Evaluation of quality of place should, if possible, follow the principle of prudent pragmatism by putting into as much implementation as possible, while minimizing the number of “immeasurable” elements. Quality of place on a national, regional, and urban scale is generally evaluated using statistical data from governmental statistic agencies. For instance, in their evaluation on China’s city quality, Zhang et al.^18^ constructed a comprehensive rating system, including indicators in four dimensions: personal consumption services, public services, health environment, and basic structure. Gu and Dong^19^ selected the five dimensions including education, medicine, culture, transportation and ecology, choosing such indicators as number of higher education institutions per 10,000 people, number of hospitals and health centers per 10,000 people, number of public library collections per 10,000 people, number of taxis per 10,000 people, and greening rate in per square meter of built area. Most urban planning and design scholars, who evaluate quality of place with a focus on macro elements such as population density, land use mix, public transport accessibility and traffic distance, also use governmental statistical data. These studies provide only an instructive and unspecific view. Little literature measures people’s subjective feeling about quality of place using questionnaires. Malkina-Pykh and Pykh^20^ for example, evaluated quality of place through people’s perception of the place, satisfaction with their life and work, and sense of happiness. However, perception occurs on an individual level, and with different preferences and different worldviews, there may be problems with data reliability^12^. With the rise of mobile phone signaling, POI data, Internet location service data, street view images and other big data and extensive application of machine learning, studies on quality of place in recent years have been deepened into neighborhood and other micro level. Recent studies have also pointed out that the perspective from which street view images are captured may affect the measurement of street perception. For example, Rui^21^ shows that driveway-based images and pedestrian-view images may produce different evaluations of walkability and visual quality, highlighting the methodological importance of viewpoint selection in streetscape assessment. For instances: Richards and Edwards^22^ quantified the road green canopy coverage in Singapore, which is more than 80%, using Google’s Street View images; also using street view images, Gong et al.^23^ estimated the sky, tree and building ratio in the city of Hong Kong; Zhang et al.^18^, based on location-based service (LBS) positioning data and POI data, studied the quality of sub-districts in Yangpu District, Shanghai; Lu et al.^24^ used mobile phone signaling and POI data to study the vibrancy of the cities of Beijing and Shanghai. In the Chinese context, street view images have also been used to investigate neighborhood-level socio-spatial issues. For instance, Yuan et al.^25^ employed street view images and deep learning techniques to examine how street-level built environment is associated with urban poverty in Guangzhou, demonstrating the value of human-scale visual indicators in understanding urban inequality. However, the selection of evaluation indicators for neighborhood quality has been considering only a small number of indicators such as greening level and building density, which may lead to partial evaluation results that cannot reflect the aggregate quality of neighborhood spaces^3^.

In summary, with the arrival of the post-materialist and post-industrialization era, quality of place has gradually become a research hotspot in urban studies field, with great progress delivered in both fields of indicator construction and evaluation/measurement, which provides valuable reference and experience for this study. However, the existing research still has limitations in some aspects that need to be addressed: (1) Research on quality of place is still dominated by national, regional and urban scales, and that on small scales, such as neighborhoods, is limited to case study of individual cities, without small-scale exploration across a larger area. (2) Existing evaluation data about quality of place still rely on governmental statistical data, failing to reflect the description of built environment in indicators established, and thus neglecting user experience, lacking objectiveness, and failing to accurately reflect the connotation of quality of place. (3) In evaluating neighborhood quality using street view images and other big data, scholars pay attention only to a few elements in individual cities or neighborhoods and focus on the “hard” elements in the built environment of the neighborhood, without “soft” elements to reflect tolerance, culture environment and creativity, making it difficult to fully reflect neighborhood quality.

Given the above discussion, this study tires to, based on the theory of quality of place as proposed by Florida, take into account the assessment criteria of quality of place adopted in the existing research to construct an evaluation system for quality of place. This system will be forged from six dimensions: Built Environment, Cultural Atmosphere, Creativity, Inclusiveness, Brand image, and Business Environment; and it would measure both the “soft” and “hard” environments of cities. By virtue of Baidu Street View images and other multi-source big data, the elements at the neighborhood level will be quantitatively described, and the quality of place of major cities in China will be evaluated by taking neighborhoods (which, in this study, refer to sub-districts, a kind of administrative agencies for neighborhoods in China) as the evaluation unit. In order to differentiate from the existing research on assessing the quality of place with a nation or a city as the unit, this study defines as Neighborhood Quality for assessing the quality of place with a neighborhood (sub-districts) as the evaluation unit.

The marginal contributions of this study are as follows: (1) A methodology has been developed that can describe urban built environment on a large scale, and can simulate the user’s point of view to describe the urban environment objectively and quantitatively on a large scale, thus remedying one of the shortcomings of the existing research—the lack of quantified description of the built environment. (2) An evaluation system for quality of place has been constructed that is applicable to China and can measure both “soft” and “hard” environments of cities, thus correcting the deficiency of insufficient measurement of “soft” environment in China’s assessment and exploration of quality of place. (3) By conducting urban research with neighborhoods as unit, this study advances the exploration of quality of place to the research of “Neighborhood Quality”, so it is an attempt of microscopic urban exploration. (4) A full evaluation framework for sub-district quality has been proposed and a rapid and accurate evaluation method has been established using mass data, which provide an important theoretical support for improvement of neighborhood quality planning and design at a human-scale, urban renewal, and optimization of urbanization policies. (5) Big data technologies, including deep learning and street view images, have conducted assessments of 5829 major neighborhoods across China, providing urban planners and policymakers with critical insights into the current status of urban development.

## Construction of an indicator system for neighborhood quality

Research on neighborhood quality in this paper is grounded in the broader literature on quality of place. Existing studies suggest that the attractiveness of place depends not only on physical urban conditions, but also on cultural, social, and economic environments that support everyday life and interaction^11,12,26^. At the neighborhood scale, these factors are experienced more directly, since neighborhoods constitute the most immediate human-scale units in which residents perceive and use urban space^4^.

However, Florida’s framework was developed mainly for Western cities and at the city level. Direct application to neighborhood-level analysis in China is therefore limited. Chinese cities exhibit strong regional heterogeneity, a distinctive sub-district-based administrative structure, and uneven development between physical construction and social-cultural conditions. Moreover, neighborhood-level assessment requires indicators that are both spatially explicit and operational at a finer scale. For these reasons, this study adapts rather than directly adopts the quality-of-place framework, combining it with the institutional and spatial characteristics of Chinese cities.

Following this approach, neighborhood quality is understood as the combined outcome of two types of factors: the hard environment and the soft environment. The hard environment refers to material and functional conditions, such as the built setting and the availability of commercial and service facilities. The soft environment refers to cultural, social, and innovative attributes, including cultural atmosphere, inclusiveness, and creativity. Based on this distinction, and drawing on Trip’s^15^ reorganization of quality-of-place indicators, this study constructs a six-dimensional framework consisting of Built Environment, Cultural Atmosphere, Creativity, Inclusiveness, Brand Image, and Business Environment. Among these, Built Environment and Business Environment mainly capture the hard environment, while Cultural Atmosphere, Creativity, and Inclusiveness reflect the soft environment. Brand Image represents the external recognition and accumulated influence of neighborhood qualities.

Recent studies have also highlighted the value of multi-dimensional and fine-grained neighborhood assessment based on street view imagery. For example, Qiu et al.^27^ integrated street view images and multi-task learning to predict neighborhood wealthiness at a fine spatial scale, showing that multiple perceptual dimensions can improve the understanding of intra-urban heterogeneity. This line of research further supports the need to move beyond single-indicator approaches when evaluating neighborhood quality.

On this basis, and in combination with existing studies on local comfort, urban culture, and neighborhood-scale development, an indicator system for neighborhood quality is constructed. The rationale for each dimension is described as follows.

### Built environment

The built environment provides the material foundation of neighborhood quality. In the quality-of-place literature, urban form, accessibility, and environmental conditions are regarded as key determinants of livability and spatial functionality^11,12^. At the neighborhood level, these factors directly shape residents’ daily activities and perceptual experiences.

In Chinese cities, the built environment at the sub-district level has particular significance. Sub-districts are basic units of urban planning and governance, and neighborhoods within the same unit often exhibit relatively similar development conditions. At the same time, rapid urbanization has generated clear differences across neighborhoods in terms of density, greenness, and accessibility. These differences make the built environment an essential dimension for distinguishing neighborhood quality.

In this study, Built Environment is measured using four indicators: traffic fluency (C01), building density (C02), green visible index (C03), and streetscape permeability (C04). Traffic fluency reflects connectivity and mobility conditions within the neighborhood. Building density indicates development intensity and spatial concentration. The green visible index measures the proportion of visible vegetation at street level, which is closely related to environmental comfort and well-being^28^. Streetscape permeability reflects visual openness and spatial continuity, influencing perceived safety and walkability^29^. Compared with traditional macro indicators, these measures capture the built environment more directly from a human-scale perspective.

### Cultural atmosphere

Cultural atmosphere reflects the experiential and symbolic qualities of neighborhood space. In the quality-of-place literature, cultural vibrancy, leisure amenities, and historical context are important sources of place attractiveness^11,15^. At the neighborhood scale, these factors influence not only spatial use, but also residents’ sense of identity and belonging.

In the Chinese context, cultural atmosphere is shaped by both the availability of cultural facilities and the historical and natural resources embedded in specific places. Cultural heritage sites, scenic resources, and public cultural facilities are unevenly distributed across sub-districts, creating significant differences in cultural experience. Chinese studies have also emphasized that cultural atmosphere includes both material support and historical-cultural accumulation^30^.

Based on this understanding, Cultural Atmosphere is measured through three indicators: cultural facilities (C05), historical profundity (C06), and natural resources (C07). Cultural facilities, such as museums, theaters, and libraries, provide the basis for cultural activities and reflect public service provision. Historical profundity captures the presence of heritage sites and historically significant locations. Natural resources, including scenic areas and parks, contribute to both recreational and cultural experiences. Together, these indicators reflect both the material and experiential dimensions of neighborhood culture.

### Creativity

Creativity reflects the capacity of neighborhoods to support knowledge production, innovation, and talent concentration. In quality-of-place research, technology, talent, and creative activities are considered key factors shaping urban vitality^11,15^. At the neighborhood scale, creativity is closely linked to the spatial distribution of institutions that generate and diffuse knowledge.

In Chinese cities, universities, research institutes, and key laboratories tend to cluster spatially, forming localized innovation hubs. These areas usually exhibit stronger knowledge spillovers, higher levels of human capital, and more dynamic socio-economic interactions. As a result, the presence of such institutions provides a practical way to capture neighborhood-level creativity.

In this study, Creativity is measured using three indicators: universities (C08), national key laboratories (C09), and scientific research institutions (C10). Universities are important centers for talent cultivation and knowledge dissemination. National key laboratories represent advanced research capacity and technological strength. Scientific research institutions contribute to applied research and the transformation of scientific achievements. Together, these indicators provide a spatially observable representation of creativity.

### Inclusiveness

Inclusiveness refers to the extent to which neighborhood space supports equitable access, social diversity, and people-oriented development. In the quality-of-place literature, tolerance and diversity are considered important conditions for sustainable urban development^11,26^. At the neighborhood level, inclusiveness is reflected in both spatial resource allocation and the ability to accommodate different social groups.

This issue is particularly important in China, where rapid urbanization has been accompanied by large-scale population mobility. The provision of inclusive public services and the integration of diverse populations have become key challenges in urban development. At the sub-district level, inclusiveness is reflected in access to facilities, the distribution of resources, and the availability of basic services.

Accordingly, Inclusiveness is measured through three indicators: fairness and justice (C11), respect for diversity (C12), and people-oriented services (C13). Fairness and justice reflect the balance and accessibility of spatial resources. Respect for diversity captures the presence and integration of different population groups. People-oriented services are measured through the availability of healthcare, living services, and educational and cultural services, which directly affect residents’ well-being^31^.

### Brand image

Brand image reflects the external recognition and symbolic influence of neighborhoods. Although it is not always treated as a separate dimension in early quality-of-place studies, it is related to the concept of “impact,” which concerns the extent to which places attract attention and generate broader influence^11^.

In Chinese cities, the importance of neighborhood brand image has increased with the development of digital platforms and online information dissemination. The reputation of neighborhoods is shaped not only by local experience, but also by search behavior, media exposure, and public recognition. Neighborhoods with strong commercial, cultural, or historical identities tend to attract more attention and accumulate higher symbolic value.

To capture this dimension, Brand Image is measured through two indicators: breadth (C14) and depth (C15). Breadth reflects the level of public attention, measured by the frequency of related entries in major search engines. Depth reflects the degree of recognition, measured by whether the neighborhood has received relevant national honors. This distinction follows the idea that place influence includes both visibility and recognition^32,33^.

### Business environment

Business Environment reflects the economic and commercial functions embedded in neighborhood space. In quality-of-place research, the availability of services and amenities is an important factor influencing residential choice and urban attractiveness^11,34^. At the neighborhood level, the business environment directly affects consumption convenience and the intensity of daily activities.

In Chinese cities, sub-districts are important units for organizing commercial activities. With the expansion of service industries and consumption spaces, neighborhoods differ significantly in terms of commercial vitality, diversity, and scale. These differences are closely related to economic development and functional specialization.

In this study, Business Environment is measured using three indicators: commercial vitality (C16), business diversity (C17), and market size (C18). Commercial vitality reflects the intensity of economic activity, proxied by nighttime light data. Business diversity captures the variety of commercial formats and services. Market size reflects the scale of economic activity, measured by the number of commercial entities. Together, these indicators describe both the intensity and structure of neighborhood-level economic activity.

The indicators are summarized in Table 1. Due to space reasons, the measurement process of the tertiary indicators is not illustrated in the main body of the text. The details of them are shown in Appendix 1.

**Table 1:** Summary of tertiary indicators and data sources.

Primary indicator	Secondary indicator	Tertiary indicator	Data source
**(**B1) Built Environment	C01 Traffic fluency	C011 Sidewalk ratio	Deep learning and Baidu street view images
		C012 Roadway ratio	
		C013 Sidewalk fluency	
		C014 Roadway fluency	
		C015 Completeness of transportation infrastructure	
	C02 Building density	C021 Building ratio	
	C03 Green visible index	C031 Plant ratio	
	C04 Permeability of streetscape	C041 Sky ratio	
		C042 Street wall ratio	
(B2) Cultural atmosphere	C05 Cultural facilities	C051 Museums (number)	Reverse geocoding of The Directory of all Museums in People’s Republic of China^a^
		C052 Theaters and cinemas (number)	POI
		C053 Public libraries (number)	
	C06 Historical profundity	C061 Modern cultural heritage sites (number)	Reverse geocoding processing of the list of major historical and cultural sites protected at the national level (Batches 1 to 7)^b^
		C062 Historical and cultural preservation sites (number)	
	C07 Natural resources	C071 National AAAAA Class Scenic Spot (number)	Reverse geocoding of A-level scenic spots list from http://www.bytravel.cn/
		C072 National AAAA Class Scenic Spot (number)	
		C073 National AAA Class Scenic Spot (number)	
		C074 National AA Class Scenic Spot (number)	
		C075National A Class Scenic Spot (number)	
		C076 Greenland parks (number)	POI
(B3) Creativity	C08 University	C081 Project 985 (number)	Reverse geocoding processing of the list of universities and colleges in China in the 2016^c^
		C082 Project 211 (number)	
		C083 Other universities (number)	
	C09 National key laboratory	C091 Enterprise national key laboratories (number)	Reverse geocoding processing of the laboratory list in the 2016 International Key Laboratory Annual Report^d^
		C092 National & local joint key laboratories (number)	
		C093 Key laboratories in universities and colleges (number)	
	C10 Scientific research institution	C101 Chinese Academy of Sciences research institutes (number)	Reverse geocoding processing of the research institutes list published by the University of Chinese Academy of Sciences^e^
(B4) Inclusiveness	C11 Fairness and justice	C111 Balance of sidewalk and roadway routes	Deep learning and Baidu street view images
		C112 the percentage of the disadvantaged groups population	The national sixth and seventh population census
		C113 Transportation hubs (number)	POI
	C12 Respect for diversity	C121 Functional diversity	
		C122 the percentage of the outlanders’ population	The national sixth and seventh population census
	C13 People oriented	C131 Level I hospitals (number)	Reverse geocoding processing of the hospital list in https://m.99.com.cn/
		C132 Level II hospitals (number)	
		C133 Level III hospitals (number)	
		C134 Other hospitals (number)	
		C135 Living services (number)	POI
		C136 Science, education and cultural services (number)	
(B5) Brand image	C14 Breadth for brand image	C141 Number of related entries in Baidu search engine in the past 10 years	Baidu search engine
		C142 Number of related entries in Tencent search engine in the past 10 years	Tencent search engine
		C143 Number of related entries in 360 search engine in the past 10 years	360 search engine
	C15 Depth for brand image	C151 Whether it has received national honors	Reverse geocoding processing of the list of Famous Historical and Cultural Cities, Towns and Villages^f^
(B6) Business environment	C16 Commercial vitality	C161 Level of economic development	NPP-VIIRS night light remote sensing data
	C17 Business diversity	C171 Business diversity	POI
	C18 Market size	C181 Number of merchants	

^a^URL Source: https://wwj.zhengzhou.gov.cn/wwml/3194642.jhtml.

^b^URL Source: https://www.maigoo.com/goomai/151573.html.

^c^URL Source: http://www.moe.gov.cn/srcsite/A03/moe_634/201606/t20160603_248263.html.

^d^URL Source: https://www.most.gov.cn/xxgk/xinxifenlei/fdzdgknr/zfwzndbb/201805/P020180521576150932136.pdf.

^e^URL Source: https://www.ucas.ac.cn/site/3.

^f^URL Source: https://baike.baidu.com/item/%E4%B8%AD%E5%9B%BD%E5%8E%86%E5%8F%B2%E6%96%87%E5%8C%96%E8%A1%97%E5%8C%BA/17353170?fr=aladdin.

## Methodology

### Neighborhood division methods

Neighborhoods are an important element in urban construction, planning and design. Different disciplinary domains have different approaches to defining and dividing neighborhoods, which can be roughly divided into five categories: (1) To divide neighborhoods in reference to urban road systems by taking urban roads as the boundary, trying not to cross railroads, urban highways, and traffic arterials^35^. (2) To delineate neighborhoods based on natural environmental features, respecting the city’s inherent boundaries and prioritizing natural elements such as rivers, mountains, and green spaces as dividing lines^36^. (3) Demographic neighborhoods. The census in the United States is based on neighborhoods as a statistical unit. For demographic neighborhoods, there are no specific requirements for the delineation of neighborhoods. Generally, the natural environment or social characteristics of a city will be used for division^37^. (4) To divide based on buildings and land parcel ownerships. This method is usually used to describe the division of large-scale real estate projects. For instance, the real estate project of Venice Water City in Pukou District, Nanjing, China. (5) Administrative division neighborhoods, which are fundamental units of county and city administration and planning. In China, these include county-level townships and towns, as well as sub-districts within city districts. The administrative organs of each units have a perfect management system and a statistics system for basic information data. They are the basic constituent unit of urban structures, planning and development. When carrying out urban construction in China, city administrators also tend to take sub-districts as a unit in such tasks as epidemic control, infrastructure layout, transportation hub layout, cityscape optimization, etc. Therefore, there is often a high degree of correlation and similarity between communities in the same sub-district.

Given that the quality of a neighborhood is the result of long-term accumulation of individuals or collectives in their social activities, such quality contains the local historical and cultural accumulation, the evolution of architectural styles and the cultivation of local customs. Therefore, neighborhoods are non-replicable, with non-tradable diversity as well as historical and cultural uniqueness; they contain a large amount of “soft” environment contents^38^. Nevertheless, the methods of road division, natural environment division, building division cannot reflect the consideration of soft environment. The demographic neighborhood division method is from the human perspective and can reflect the accumulation of social activities, but it does not fit the actual situation of China. This study takes China as the research object, where urban sub-districts are the basic unit for city administrators to carry out urban construction. As a result, there is similarity and correlation between the community units under the same urban sub-districts. Development under long-term unified administration gives a high degree of correlation to the social activities, atmosphere evolution, and built environment within the same urban sub-district. Therefore, this study adopts the standard of China’s administrative division as the criterion for the division of urban neighborhoods, while choosing urban sub-districts as the basic research object for neighborhood quality.

### Evaluation method of neighborhood quality

**Fig. 1:**
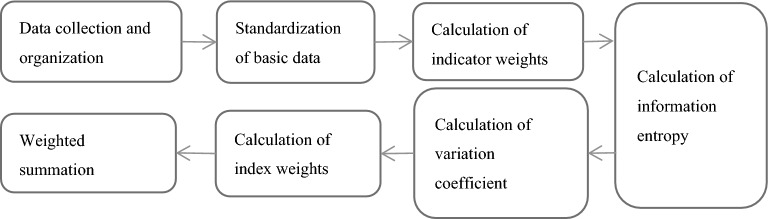
Overview of evaluation steps via the entropy weight method.

In this study, the entropy weight method is adopted to construct a comprehensive evaluation model for neighborhood quality. This method can assign weights according to the degree of dispersion between indicators, thus avoiding human’s subjective judgments and circumventing the overlapping of information between indicator variables. This method has been widely implemented in the comprehensive evaluation research in various fields. The evaluation of neighborhood quality using the entropy weight method can be divided into seven steps: data collection and organization, standardization of basic data, calculation of indicator proportion, calculation of information entropy, calculation of variation coefficient, calculation of index weights, and weighted summation, as shown in Fig. 1:

Given the extensive application of the entropy method in related studies, we focus here on its implementation, while detailed formulas are omitted for brevity. (Detailed steps can be found in Appendix 6.)

### Methods of acquiring the evaluation data

In this paper, we design a mining method to obtain neighborhood-level data using deep learning, Baidu Street view images, POI data, and the use of Baidu’s inverse geocoding tool. Among them, the deep learning and Baidu Street view is able to obtain mass but small-scale data at the neighborhood level, which is also the main data source used to measure the quality of neighborhoods in this paper. In addition, considering that Baidu Street view images and deep learning method is unable to measure the number and types of functional units in a neighborhood, this paper uses POI data and Baidu’s inverse geocoding tool to make up for these shortcomings.

#### Deep learning and Baidu street view images

(1) Mining of street view images

In the existing literature, street view data primarily comes from Google Street View (GSV) and Baidu Street View (BSV). BSV, which primarily serves Chinese users, has collected street panorama data from over 650 cities in China. Furthermore, Baidu Map offers a free download platform, and the BSV API can be accessed using Python software for online retrieval and batch downloading. Each Baidu Street View image contains complete metadata including longitude, latitude, shooting angle, capture timestamp, and the administrative sub-district information corresponding to the shooting location.

This study uses Python software to access the BSV API and create a programme to capture city street view images based on street latitude and longitude sampling points. As a result, street view images from districts in 5829 major sub-districts in China were collected. The methodology is presented in the following steps:

**The first set** of neighborhood network data was obtained from the Open Street Map (OSM) community. The initial OSM road network data covered a wide range of road types, including primary, secondary, and tertiary roads, residential and service roads, cycleways, paths, walks, pedestrian streets, connecting roads, rural roads, track grades, motorways, and elevated highways.

**Fig. 2:**
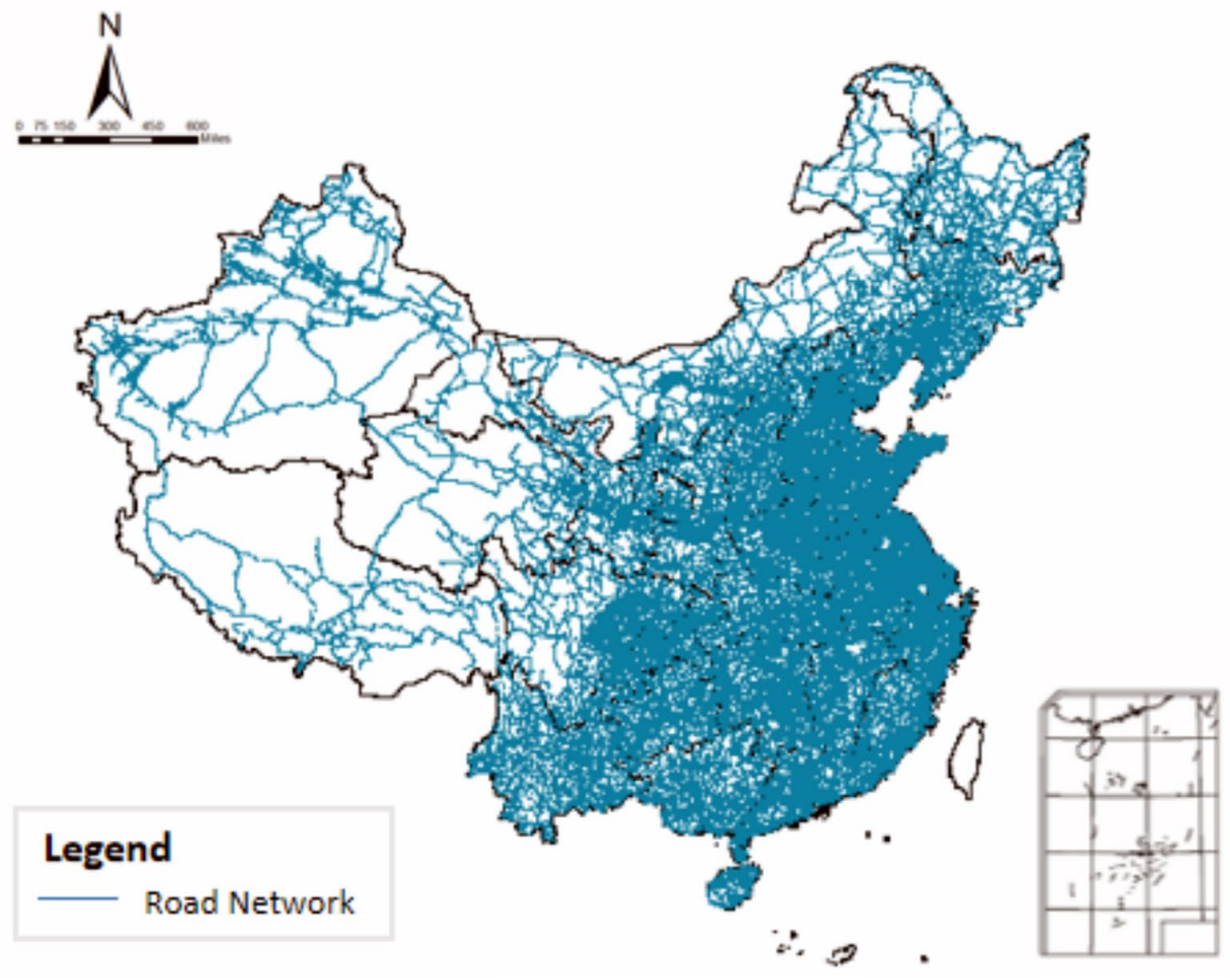
Schematic diagram of the OSM road network in 2016.

ARCGIS software helps visualize the distribution of the national road network (refer to Fig. 2). There is a strong correlation between the density of China’s road network and regional economic development levels. The eastern and central regions are more economically developed, with denser road networks than the western region. Given that OSM road network data is more accurate on high grade roads^39^, five categories of higher level roads were selected to represent them: primary roads, secondary roads, tertiary roads, residential roads, and service roads.

**The second step** is to generate street view images collection points. This study employs ARCGIS to generate a large number of collection points at random along the established road network, building on the method proposed by Li et al.^28^. A minimum distance of "100 m" is established to ensure diverse collection points on each road. This method is widely recognised and referenced in scholarly research^22^.

**Fig. 3:**
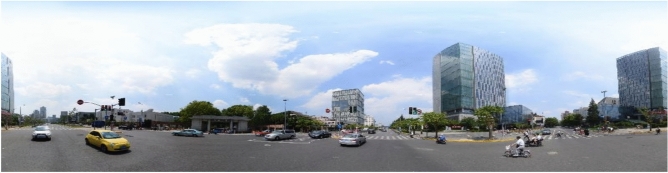
Full view of the street view.

**Fig. 4:**
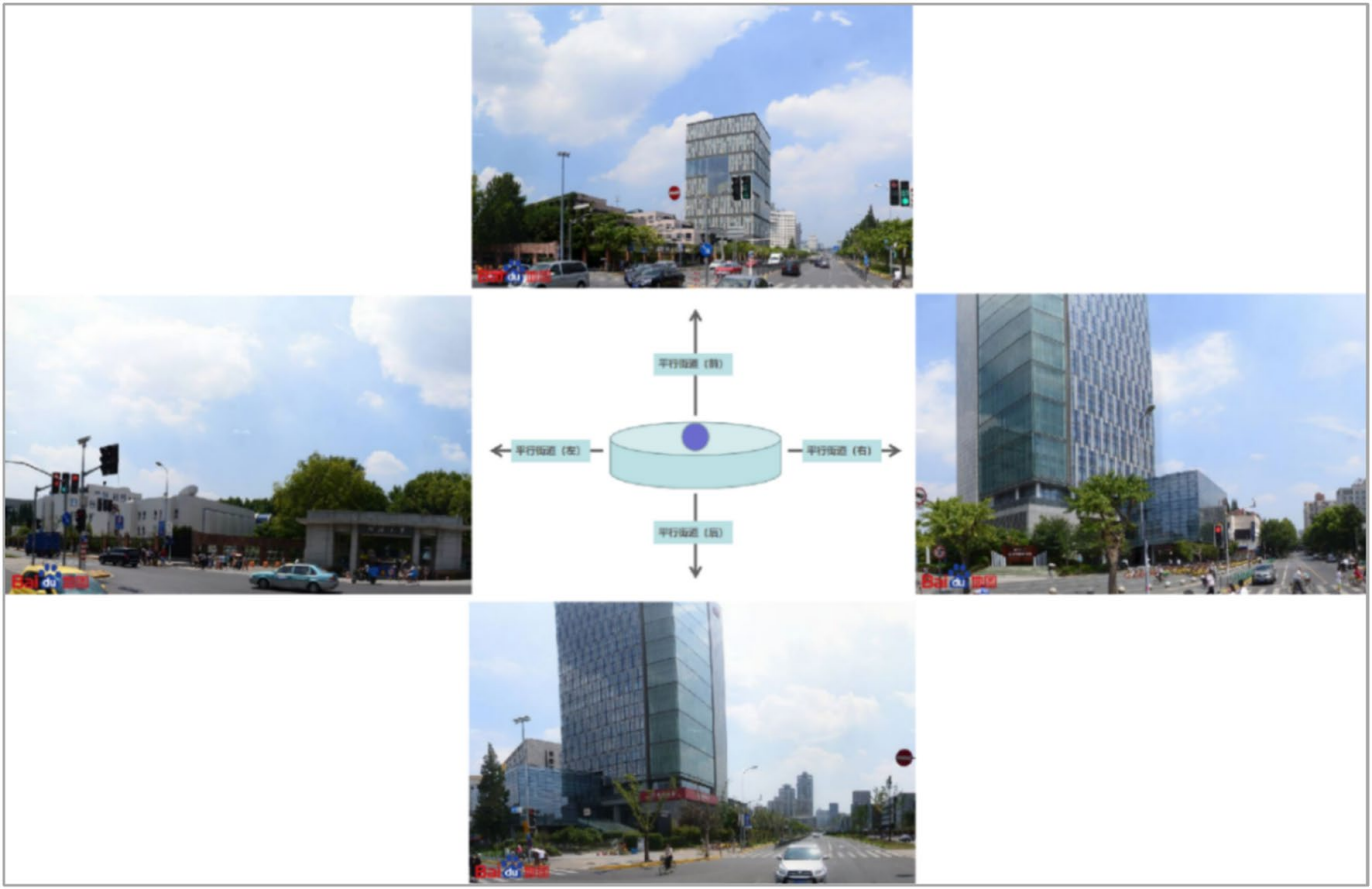
Angle view of street view images.

**The third step** is to collect Street View images. To obtain Baidu Street View images, Python software is used to access the Baidu Street View API and collect images for all collection points in a batch. To align with human visual perception, this study adopts existing scholarly practices, setting the vertical viewing angle range of street view images from 10° to 45°^40^. Full-angle image capture, due to the spherical shooting technique used in the Baidu Street View capture vehicle, can result in image deformations (refer to Fig. 3), potentially causing errors in subsequent recognition tasks. As a result, this paper sets the horizontal direction range (fov) value to 90°, collecting street view images at 0, 90, 180, and270° for each collection point. This approach ensures comprehensive 360° coverage of street view images from each collection point and avoids image distortion (refer to Fig. 4).

Also consider that in China, the green vegetation in the area north of the Yellow River generally withers in winter. If the time and location of the street view acquisition happen to be in this situation, it will produce errors in the recognition of plants, sky and other elements in the scene. To eliminate these biases, this research removes images whose locations are cities north of the Yellow River and whose collection time is winter (December–February) (about 0.31% of the total number of images). In the end, using the aforementioned methodology, over 7.8 million Baidu Street View images were collected from 5829 main sub-districts across 232 cities in China, with a resolution of 860 × 573 pixels.

To reduce seasonal bias, winter images in northern cities were excluded. However, as the street view data were obtained from an open platform, the acquisition time is not fully synchronized across locations. The images used in this study therefore vary to some extent in both year and season.

This temporal heterogeneity is difficult to avoid in large-scale street view studies covering multiple cities (e.g^4^.,). In principle, differences in acquisition time may affect certain visual features, particularly those related to vegetation conditions, lighting, and street activity. As a result, temporal variation may introduce additional measurement noise in cross-regional comparisons.

That said, the likelihood of systematic bias is relatively limited for two reasons. First, the analysis is conducted at the neighborhood level based on a large number of observations, which helps average out idiosyncratic variation in individual images. Second, the key indicators used in this study are primarily derived from relatively stable physical elements, such as buildings, roads, and sky, which are less sensitive to short-term temporal fluctuations.

Nevertheless, temporal inconsistency remains a potential source of uncertainty. Future research could improve comparability by using street view data collected within consistent time periods or by explicitly incorporating temporal information into the analysis.

(2) Recognition of street view images

Deep learning has made it possible to conduct detailed, large-scale explorations of Street View image data^41^. Previous street space research faced challenges of high labour costs and scalability, but advanced algorithms such as image segmentation and object detection overcame them. These techniques are increasingly being used in studies of urban street features^42^, urban street recognition^43^, and street space perception^44^.

**Fig. 5:**
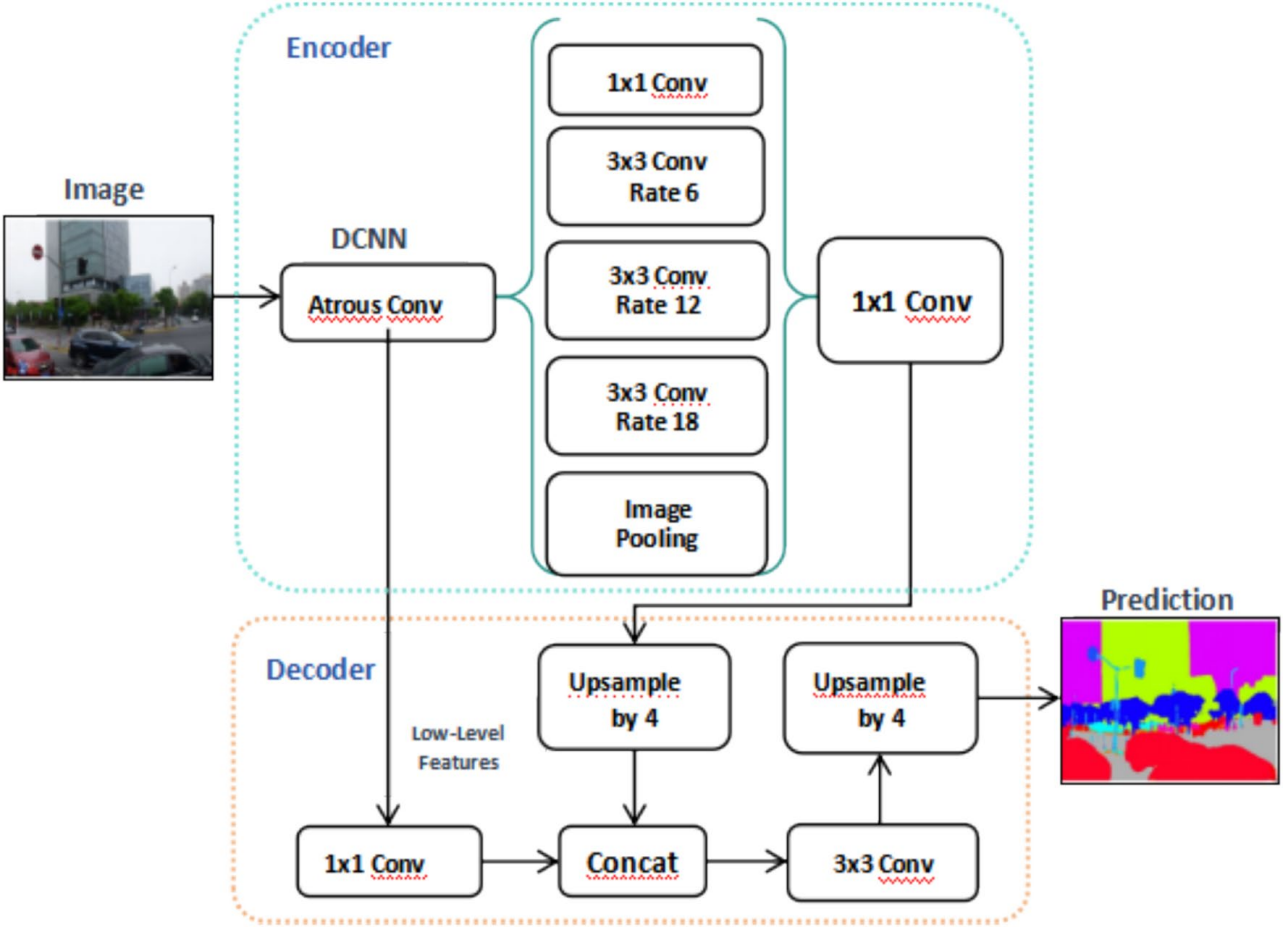
Overview of the deep lab V3 + model architecture.

All of the collected street view images were thoroughly learned using Deep Convolutional Neural Network Architecture. This allowed us to identify specific street elements and provide quantitative descriptions of neighborhood elements. The steps taken were the following:

**The initial step** involved semantic segmentation of street view images. the DeepLabV3 + model was employed to perform pixel-level semantic segmentation. DeepLabV3 + is a deep convolutional neural network that integrates atrous convolution and an encoder–decoder structure, which enables effective multi-scale feature extraction and improves segmentation accuracy, especially for complex urban scenes. Owing to its strong performance in scene parsing tasks, it has been widely adopted in studies of street-level urban environments^45^. The DeepLabV3 + model algorithm is shown in Fig. 5.

**Fig. 6:**
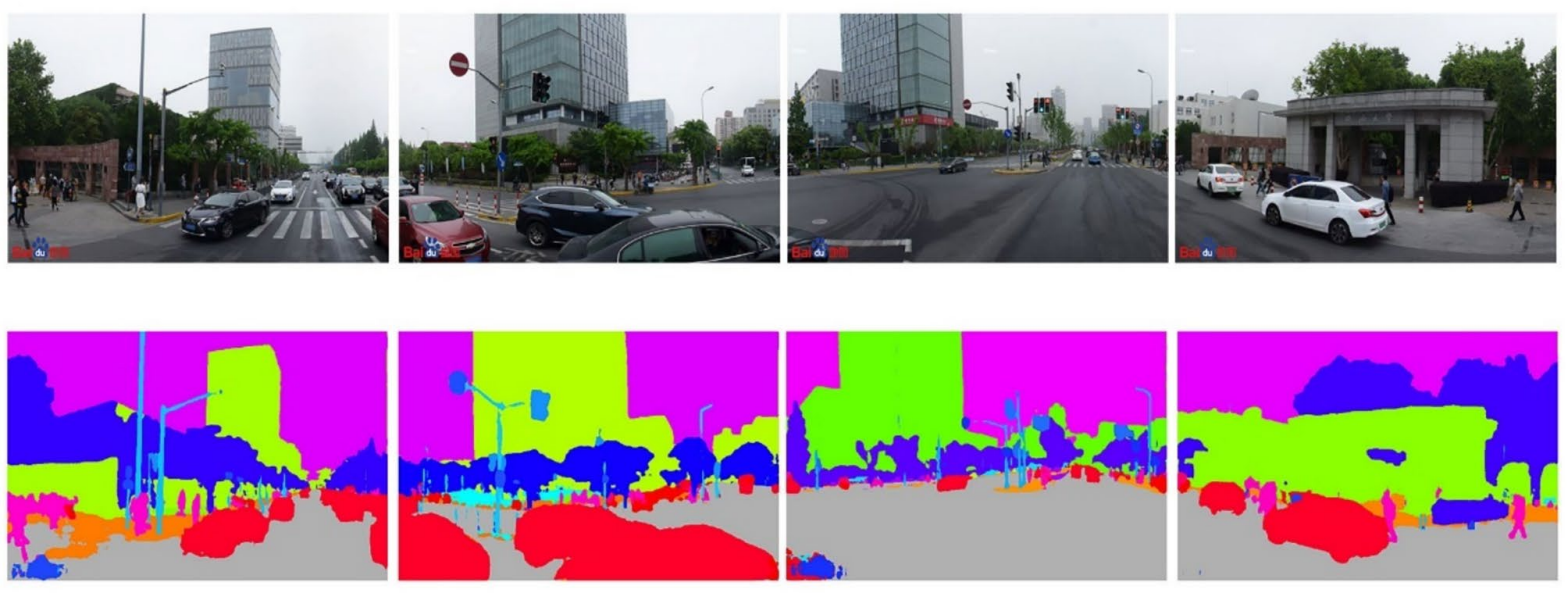
Visualization of semantic segmentation results from street view.

In object detection. The segmentation model used in this study is based on the Cityscapes dataset, which provides fine-grained annotations for 19 categories of urban street elements, including roads, sidewalks, buildings, vegetation, sky, and other typical components (see Table 2). These categories correspond closely to the physical elements required for describing neighborhood environments, making them suitable for this research. The 7.8 million Baidu Street View images were semantically segmented and color-coded to show the different parts using the Deep Lab V3 + framework and the Cityscapes dataset (Fig. 6). Following common practice in large-scale urban analytics, we adopt a pre-trained DeepLabV3 + model rather than retraining the network, as large-scale labeled street view datasets for Chinese cities are limited.

**Table 2:** Semantic segmentation analysis of cityscapes.

ID	Variable	ID	Variable	ID	Variable	ID	Variable
1	Road	6	Pole	11	Sky	16	Bus
2	Sidewalk	7	Traffic light	12	Person	17	Train
3	Building	8	Traffic sign	13	Rider	18	Motorcycle
4	Wall	9	Vegetation	14	Car	19	Bicycle
5	Fence	10	Terrain	15	Truck

**The second step** involved the quantitative description of neighborhood elements. After semantic segmentation, each street view image was converted into a labeled pixel map, where each pixel was assigned to a specific category. Based on these results, the proportion of pixels corresponding to different categories was calculated. These pixel-level statistics were then used to construct a set of visual indicators. For example, the green visible index was derived from vegetation pixels, building density from building pixels, and traffic-related characteristics from road and sidewalk pixels. Streetscape permeability was approximated using the relative proportions of sky and built elements. Through this process, unstructured image data were transformed into quantitative variables suitable for neighborhood-level analysis.

The reliability of this approach is supported by the performance of the DeepLabV3 + model on the Cityscapes benchmark dataset, which has demonstrated high accuracy in recognizing major urban elements such as buildings, roads, and vegetation^46,47^. In addition, previous studies have shown that semantic segmentation models trained on Cityscapes can be effectively applied to street view data in Chinese cities, yielding robust and consistent results in urban environmental analysis^4,28^.

Nevertheless, it should be noted that the Cityscapes dataset was originally developed based on street scenes from European cities, and applying such models to Chinese urban contexts may introduce potential domain differences. Related concerns have also been raised in recent studies on the transferability of perception datasets. For example, Rui and Cai^48^ show that datasets such as Place Pulse may produce systematic perceptual bias when directly applied to Chinese cities, particularly for dimensions that are sensitive to cultural and contextual differences. This highlights the importance of domain adaptation and localization in street view-based urban studies.

In this study, the potential impact of such transferability issues is partly mitigated in two ways. First, the analysis focuses on relatively stable and visually distinguishable physical elements, which are less sensitive to cultural interpretation. Second, the results are aggregated at the neighborhood level based on a large number of observations, which helps reduce the influence of individual recognition errors.

To enhance reproducibility, the data acquisition process, image sampling strategy, semantic segmentation workflow, and the conversion from pixel-level outputs to indicator-level variables are explicitly described above. While the use of a pre-trained model may introduce certain limitations, this approach represents a practical and widely adopted solution in large-scale urban studies where labeled data are limited.

These data will be applied to the quantitative description of indicators such as traffic fluency (C01), building density (C02), green visible index (C03), and permeability (C04).

#### Integration of POI data

While the deep learning approach used in Baidu Street View successfully distinguishes various element components from a pedestrian perspective, it falls short in identifying the number and types of functional units within neighborhoods. To bridge this gap, this study uses POI (Point of Interest) data to improve the aforementioned indicators.

POI includes point-based data from internet electronic maps. This information applies to prominent urban structures such as restaurants, tourist attractions, and hotels. It includes important information such as name, address, geographical coordinates, and category, effectively reflecting the interaction of social activities associated with these geographical entities and their locations.

For this study, each unit was classified using the POI industry codes, resulting in a total of 12,343,191 POI data entries. There are 19 categories and 137 subcategories. Using address data, the study quantifies the various units found in each neighborhood. As a result, the relevant data for the 5,829 neighborhoods under consideration was extracted and analysed.

And poi data were applied to quantitative descriptions of cultural facilities (C05), fairness and justice (C11), respect for diversity (C12), people oriented (C13), business diversity (C17), and market size (C18).

#### Utilization of inverse geocoding tools

Although POI data effectively accounts for the majority of functional units in neighborhoods, it has significant shortcomings, particularly in accurately representing museums, culturally modern units, heritage sites, hospitals, universities, and A-level tourist attractions. To address these shortcomings, this study proposes a method for more precise neighborhood unit identification that makes use of the Baidu API’s inverse geocoding feature.

**Fig. 7:**
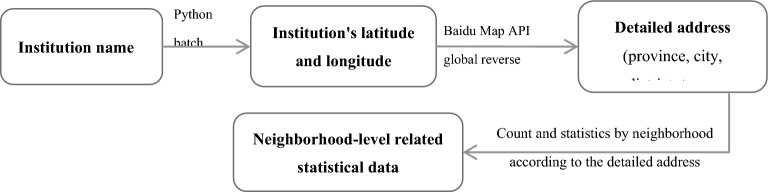
Process diagram of the inverse geocoding tool application.

Initially, a comprehensive list of venues that were underrepresented by POI data, such as museums and A-level tourist attractions, was created. Python software was then used to determine the geographic coordinates of each unit. The Baidu Map API’s global inverse geocoding tool was then used to determine detailed addresses. The final step involved calculating the quantity of each unit within neighborhoods using detailed street information from the addresses. The methodology is represented in Fig. 7.

Quantification of indicators such as cultural facilities (C05), historical profundity (C06), natural resources (C07), universities (C08), national key laboratories (C09), scientific research institutes (C10), people oriented (C13), and depth for brand image (C15) was accomplished using this method.

#### Usage of other data methods

In addition to the three main data acquisition methods described above, this paper uses NPP-VIIRS nighttime lighting data for the quantification of commercial vitality (C16) and search engine indices for the breadth for brand images (C14). The data from the sixth and seventh population census are used for the quantification of fairness and justice (C11) and respect for diversity (C12). In summary, the specific data used in this study’s evaluation indicator system for neighborhood quality mainly stem from multiple sources of big data, such as Baidu Street View images, POI data, inverse geocoding tool conversion, NPP-VIIRS nighttime lighting data, census data and search engine index. Table 1 shows the sources of the indicator data in detail.

### Selection and descriptive statistics of the research samples

This study evaluates the neighborhood quality of China by using as the research sample the 5,829 neighborhoods for which the street view images were collected. Of these 5,829 neighborhoods, 3,094 ones are located in eastern cities of China, 1,707 ones in central cities, and 1,028 ones in western cities. The samples cover 70% of prefecture-level cities in China. The process of matching the data with the research samples and the final descriptive statistics of the tertiary indicators are presented in Appendix 2.

## Results and analysis

A large number of evaluation indicators are involved in this study. Given the space constraints, the evaluation process for 10 representative neighborhood samples were selected to illustrate in Appendix 3 in detail. The weight of level-1 indicator are listed in Table 3. For details of the indicator weights at other levels, see Appendix 3.

**Table 3:** Summary of weight measurement results for neighborhood quality indicators.

Name	Built environment	Cultural atmosphere	Creativity	Inclusiveness	Brand image	Business environment
Weight	0.1191	0.2543	0.2042	0.2691	0.0660	0.0873

According to the weights of the secondary indicators in Table 3, it can be observed that the inclusiveness of a city is ranked first, which delivers the greatest influence on quality of place; the cultural atmosphere is ranked second, and the creativity is ranked third. All of these three indicators reflect the “soft” environment in quality of place, with a weight of more than 0.2. The built environment and business environment, which represent the “hard” environment in quality of place, ranked fourth and fifth, respectively. It can be seen that as the development of urbanization enters into the late stage, people’s pursuit of cities pays more attention to the “soft” environment, such as urban inclusiveness, cultural diversity, and third space. This finding is in line with the research conclusion of Zenker^49^.

**Fig. 8:**
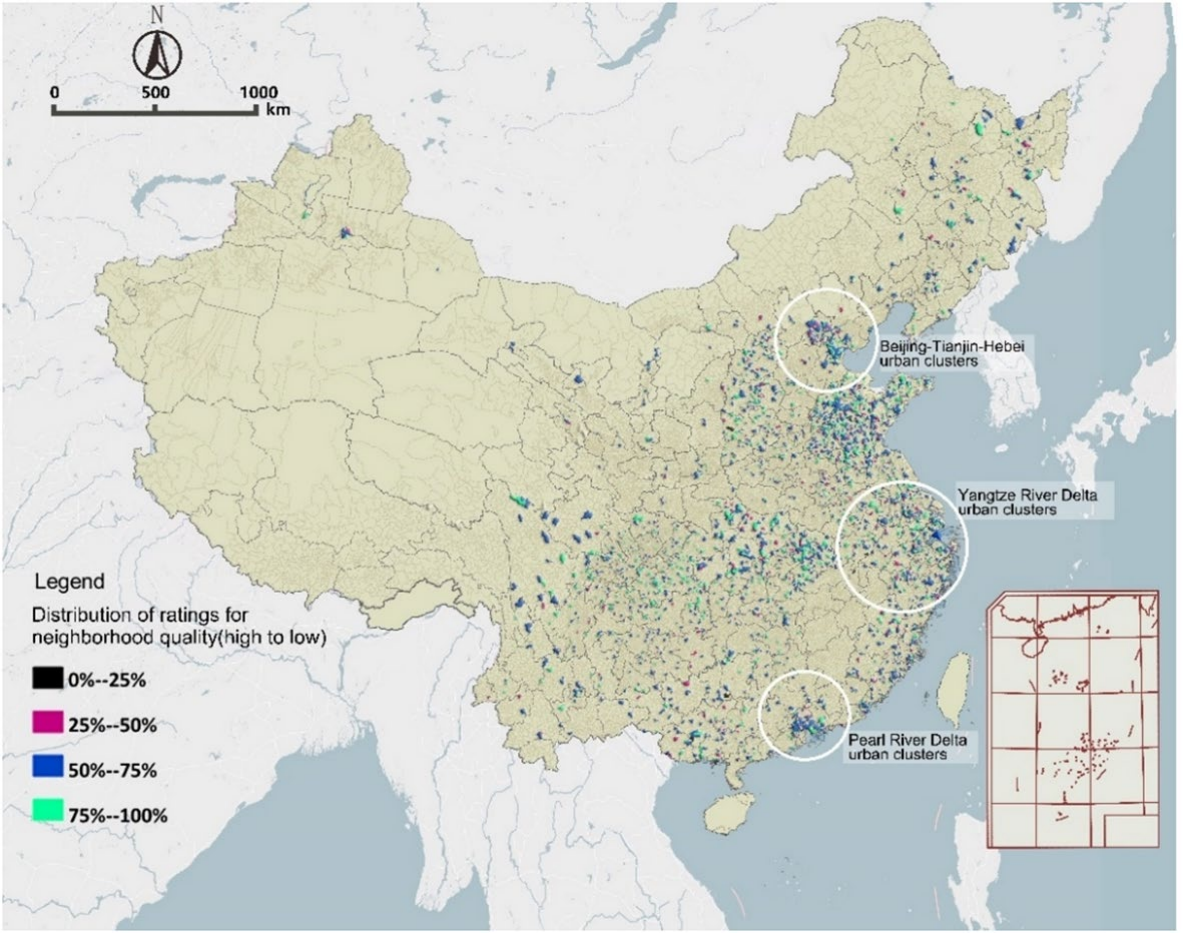
Distribution of targeted neighborhoods in China and their quality evaluation.

(2) A spatial display of the evaluation results for neighborhood quality in China is presented in ARCGIS (Fig. 8), which reveals that the neighborhood quality of China’s eastern coastal areas is remarkably higher than that of other areas. In particular, the quality of neighborhoods in urban agglomerations of the Yangtze River Delta, the Pearl River Delta and the Beijing-Tianjin-Hebei region is significantly higher than in the other regions. According to China’s statistics in 2016, the total GDP of urban agglomerations of the Yangtze River Delta (19.78% of the national GDP), the Pearl River Delta (9.83% of the national GDP) nd the Beijing-Tianjin-Hebei region (9.25% of the national GDP) accounted for 38.86% of China’s total national GDP, a number much higher than that of the other 16 national-level urban agglomerations. This demonstrates that high-quality neighborhoods in China are still frequently located in economically developed urban agglomerations. This feature is also reflected by the top 10 highest-rated quality neighborhoods as summarized in this study (Table 4). As seen in Table 4, among the 10 neighborhoods with the highest neighborhood quality ratings in China, there are 9 located in the above three urban agglomerations. Thus it can be seen neighborhood quality tends to be higher in eastern regions than in central and western China. This pattern is broadly consistent with existing studies on regional development disparities in China, which highlight the concentration of economic resources, infrastructure, and public services in eastern cities^50,51^. Similar spatial patterns have also been observed in studies on urban livability and city-level quality.

**Table 4:** Top ten high-quality neighborhoods (sub-districts) in China.

ID	Sub-district	Urban agglomeration	Score
1	Donghuamen, Beijing	Beijing-Tianjin-Hebei	0.6185
2	Nanjingxilu, Shanghai	Yangtze River Delta	0.6021
3	Xichang’anjie, Beijing	Beijing-Tianjin-Hebei	0.5968
4	Tianhenan, Guangzhou	Pearl River Delta	0.5774
5	Xujiahui, Shanghai	Yangtze River Delta	0.5752
6	Beijinglu, Guangzhou	Pearl River Delta	0.5675
7	Qianmen, Beijing	Beijing-Tianjin-Hebei	0.5601
8	Lujiazui, Shanghai	Yangtze River Delta	0.5596
9	Dazhi, Wuhan	Middle Reaches of the Yangtze River	0.5581
10	Hubin, Hangzhou	Yangtze River Delta	0.5575

**Fig. 9:**
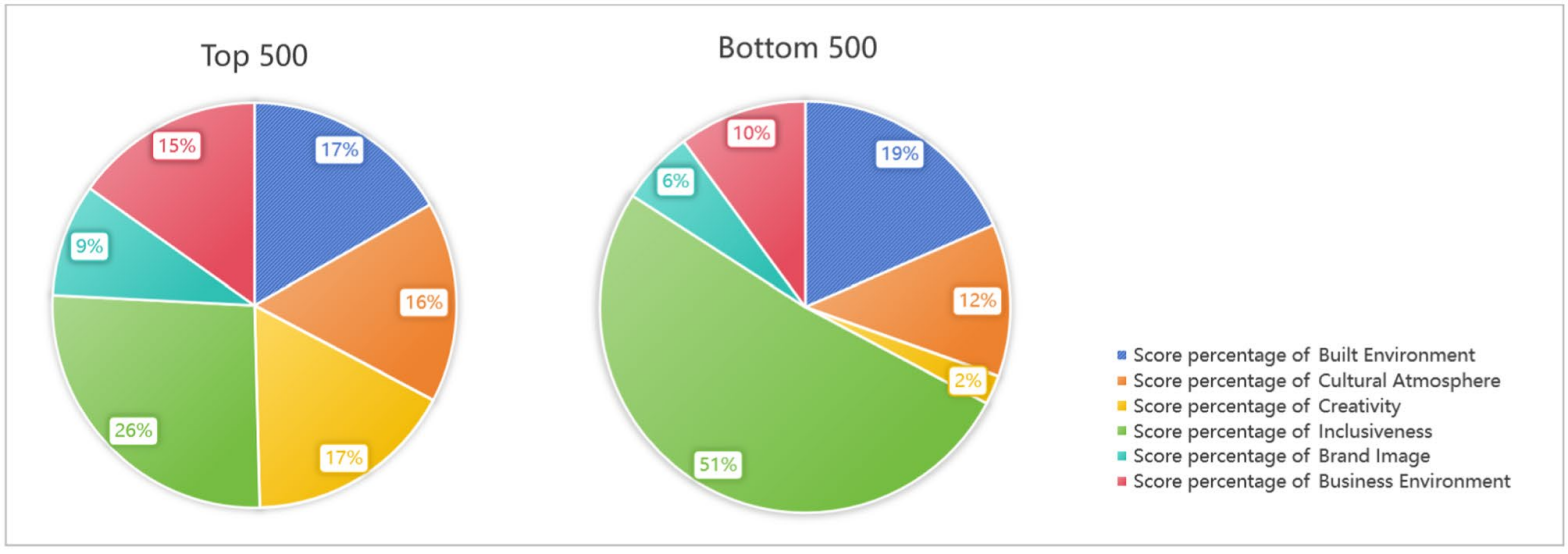
Quality score comparison among high- and low-quality neighborhoods in China (Detailed data are in Appendix 5.)

(3) In order to explore the gap between high- and low-quality neighborhoods, the quality scores of the top 500 neighborhoods and the bottom 500 ones are summarized and compared. The results are shown in Fig. 9 that the higher-quality neighborhoods are constructed in a more balanced manner in all dimensions. In contrast, the low-ranked neighborhoods demonstrate significant deviations in terms of high inclusiveness and low creativity; Interestingly, some of the lower-ranked neighborhoods exhibit relatively high inclusiveness scores. This pattern should be interpreted with caution. One possible explanation is that these neighborhoods tend to have lower population density, which may reduce competition for public resources and result in a more even distribution of basic services. To provide additional context, we further examined proxy indicators of population distribution, including population density data. The results suggest that these neighborhoods are generally associated with relatively lower levels of population concentration (The verification results are presented in the appendix.7), which is consistent with the above interpretation. However, this relationship should not be understood as a direct causal mechanism, and further investigation using more detailed demographic data would be needed. Similar patterns have been discussed in studies on urban service accessibility, which note that lower-density areas may, under certain conditions, exhibit relatively balanced access to basic services^4,26^.

In addition, Creativity-related indicators are relatively weak in lower-ranked neighborhoods, suggesting a limited presence of knowledge-producing institutions. This observation is consistent with research on the geography of innovation, which shows that talent, research institutions, and innovation resources are highly spatially concentrated in more developed urban areas^11^.

**Fig. 10:**
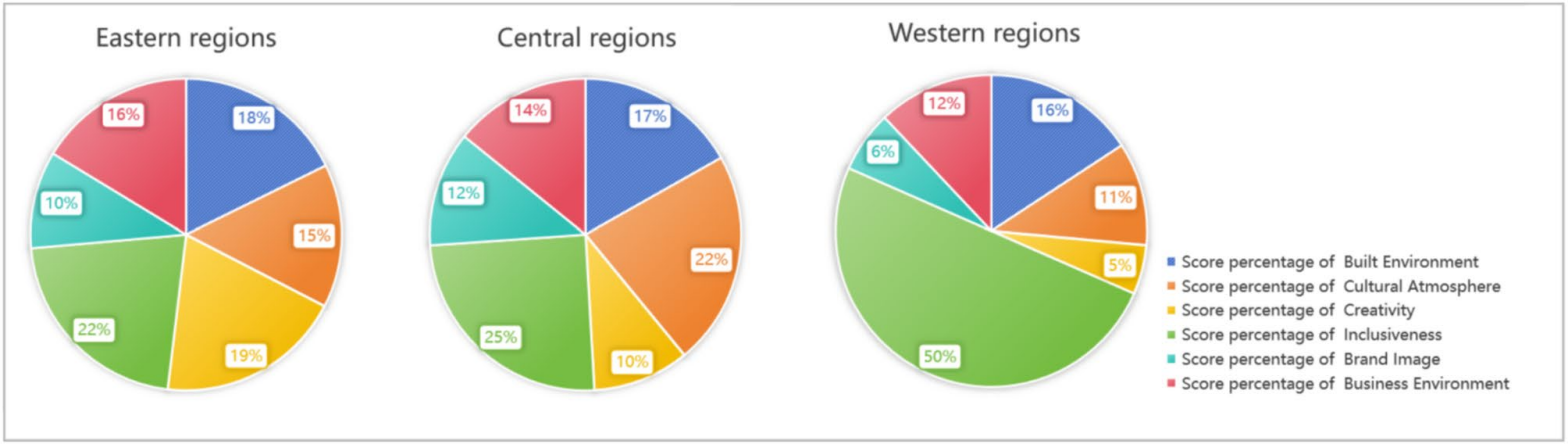
Neighborhood quality score comparison between Eastern, Central and Western cities in China (Detailed data are in Appendix 4).

(4) In order to further explore the developmental differences in neighborhood quality among regions in China, the neighborhood quality scores of cities in eastern, central and western region of China are summarized and compared. Figure 10 shows that the construction of neighborhoods in eastern cities is more balanced in all dimensions, demonstrating their advanced planning and development concepts to a certain extent. Compared with the developed areas in eastern China, the central region cities have certain advantages in the cultural atmosphere in their neighborhood construction; but they lack “creativity” somewhat. As to the neighborhoods in western cities, their construction showcases a clear deviation, as manifested in their high inclusiveness and very low creativity. Thus it can be seen neighborhood quality tends to be higher in eastern regions than in central and western China. This pattern is broadly consistent with existing studies on regional development disparities in China, which highlight the concentration of economic resources, infrastructure, and public services in eastern cities^51^. Similar spatial patterns have also been observed in studies on urban livability and city-level quality.

There are differences in the stage of development within the same city, and also in the quality of urban neighborhoods at different stages of development. To further explore the difference in quality of neighborhoods at different stages of development, this paper uses NPP-VIIRS nighttime lighting data in 2016, after ARCGIS raster processing, for calculating the average light intensity value of each neighborhood in the city. The light intensity values of neighborhoods in each group of cities are then trichotomized. Neighborhoods with upper 1/3 light intensity are defined as the “Busy areas” of the city; those with the middle 1/3 light intensity are defined as the “developing areas”; and the remaining 1/3 are defined as the “underdeveloped areas (or lagging areas)”.

**Fig. 11:**
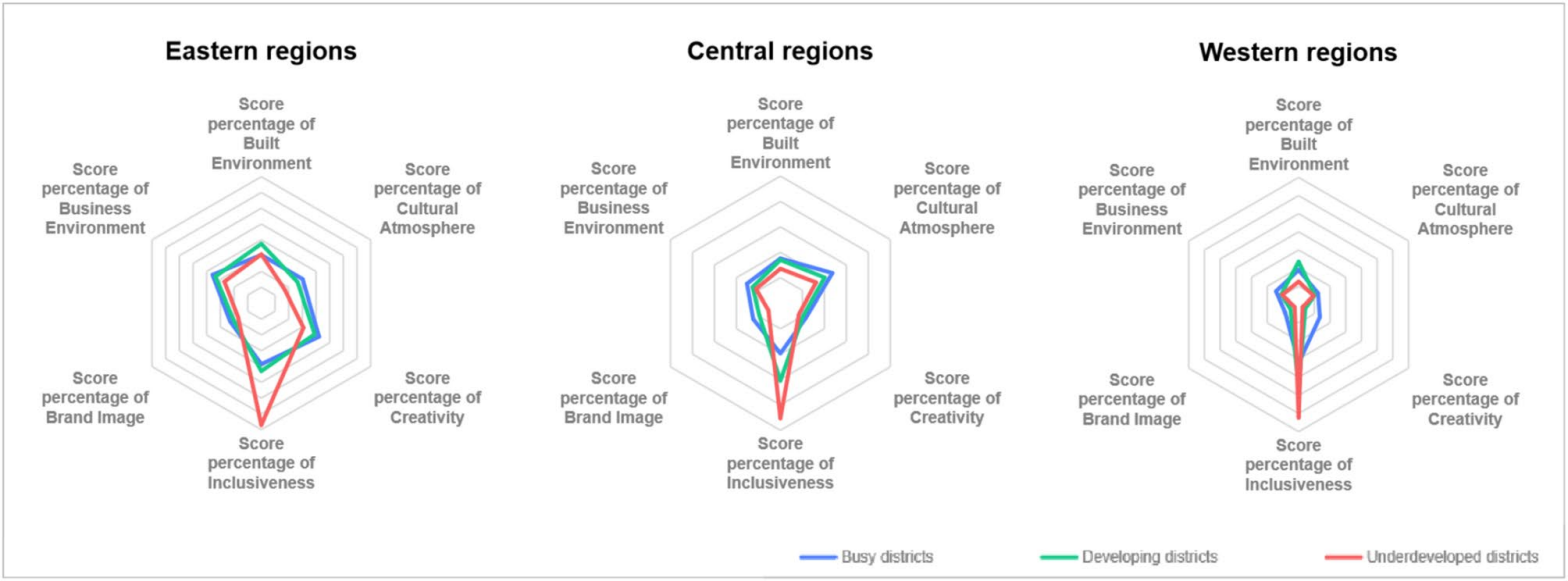
Quality score comparison among neighborhoods at different stages of development in Eastern, Central and Western Cities in China (Detailed data are in Appendix 4).

The neighborhood qualities of the three types of districts in eastern, central, and western regions are summarized and compared, as is shown in Fig. 11. It can be concluded that: (1) The neighborhoods in the busy and developing areas of eastern cities demonstrate relatively balanced performance across all quality indicators, aligning with the attributes of high-quality urban districts. (2) While central cities’ busy and developing areas exhibit a distinctive cultural atmosphere, they show a marked deficiency in creativity. (3) Western cities’ busy areas display remarkable creativity, sharply contrasting with the scarcity of innovative capacity in their developing and underdeveloped districts. This suggests that creativity in western cities is predominantly concentrated in their core urban hubs. (4) The built environment assessment scores in western cities’ developing areas are notably higher, reflecting their strategic emphasis on urban environmental development in these zones—a pattern similarly observed in eastern cities. (5) Lagging areas across all three regions demonstrate extreme levels of social inclusiveness. However, as our earlier analysis reveals, this characteristic paradoxically correlates with lower overall neighborhood quality metrics.

**Fig. 12:**
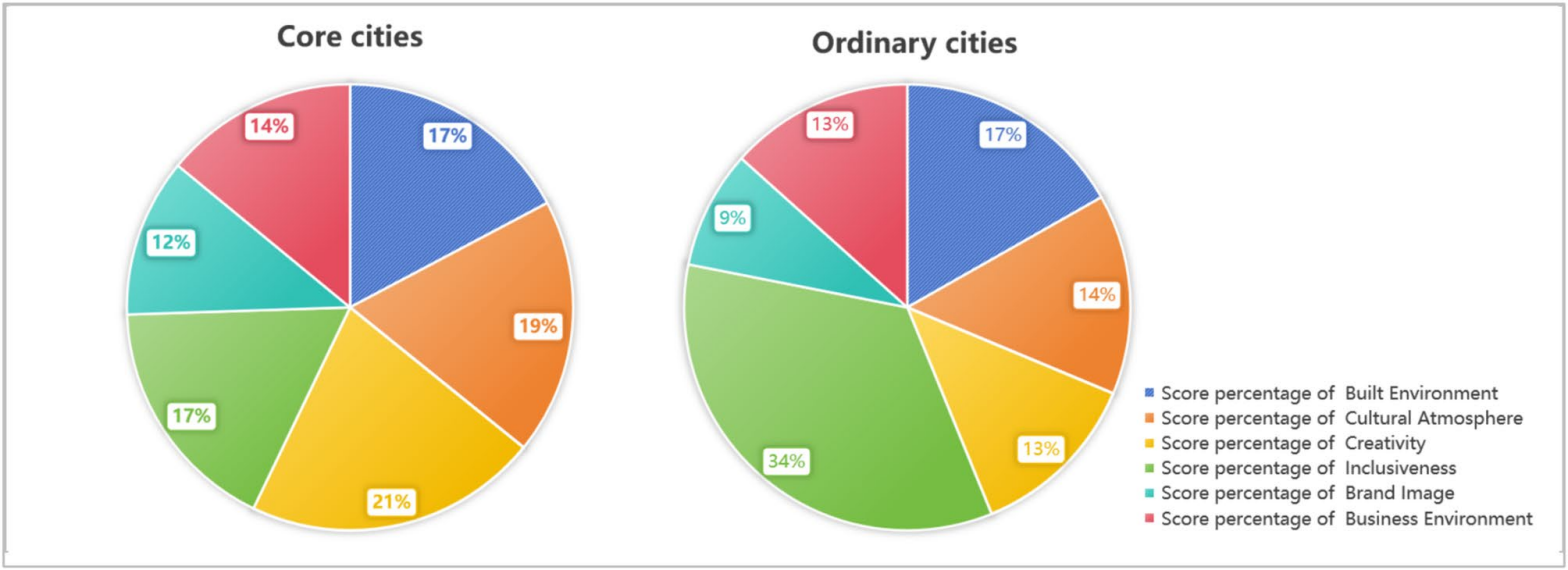
Neighborhood quality score comparison between core cities and ordinary Cities in China (Detailed data are in Appendix 4).

(5) Give that the development of China’s cities not only varies with space but also vary greatly even within the same province, we divided the cities into core cities and ordinary cities. (Detailed data are in Appendix 4.) We further explored the development difference between neighborhood quality of core cities and ordinary cities. Figure 12 shows the neighborhood quality scores of core cities and ordinary cities. It can be seen that: (1) Neighborhood quality scores of core cities are generally more balanced, which is a characteristic of high-quality neighborhoods. (2) Among all scores of core cities, creativity takes up the largest portion, demonstrating excellent creativity of such cities. (3) Between core cities and ordinary cities, difference is small in the scores of “hard” environments such as built environment and commercialization, and is significant in those of soft environment such as creativity, cultural atmosphere, inclusiveness, and brand image. Specifically, ordinary cities are scored lower in creativity, cultural atmosphere and brand image, but higher in inclusiveness.

**Fig. 13:**
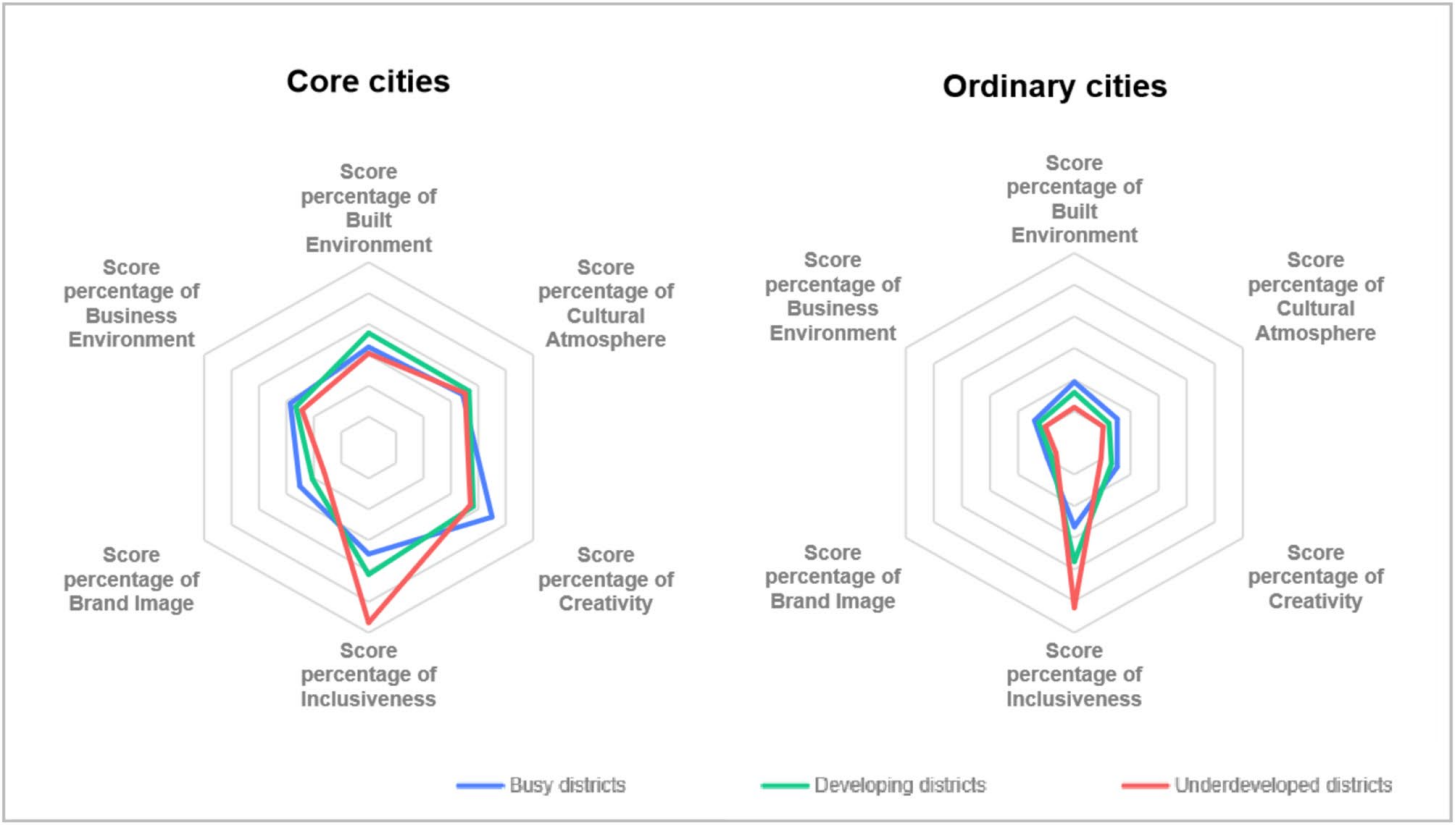
Quality comparison among neighborhoods at different stages of development in core cities and ordinary cities (Detailed data are in Appendix 4).

Figure 13 compares neighborhood quality scores of districts at different stages of development in core cities and ordinary cities. We found that (1) Neighborhood quality scores of districts in core cities, despite the stage of development, have balanced quality scores, with outstanding creativity in busy districts. (2) Developing districts have better built environment. (3) Underdeveloped districts have lower scores in brand image, but relatively balanced scores in other dimensions. (4) Districts at different stages of development in ordinary cities, however, show a clear gradient of development. Specifically, scores of built environment, creativity, cultural atmosphere, brand image and business environment descend in the order of busy districts, developing districts, and underdeveloped districts, while the score of inclusiveness ascends in this order.

**Fig. 14:**
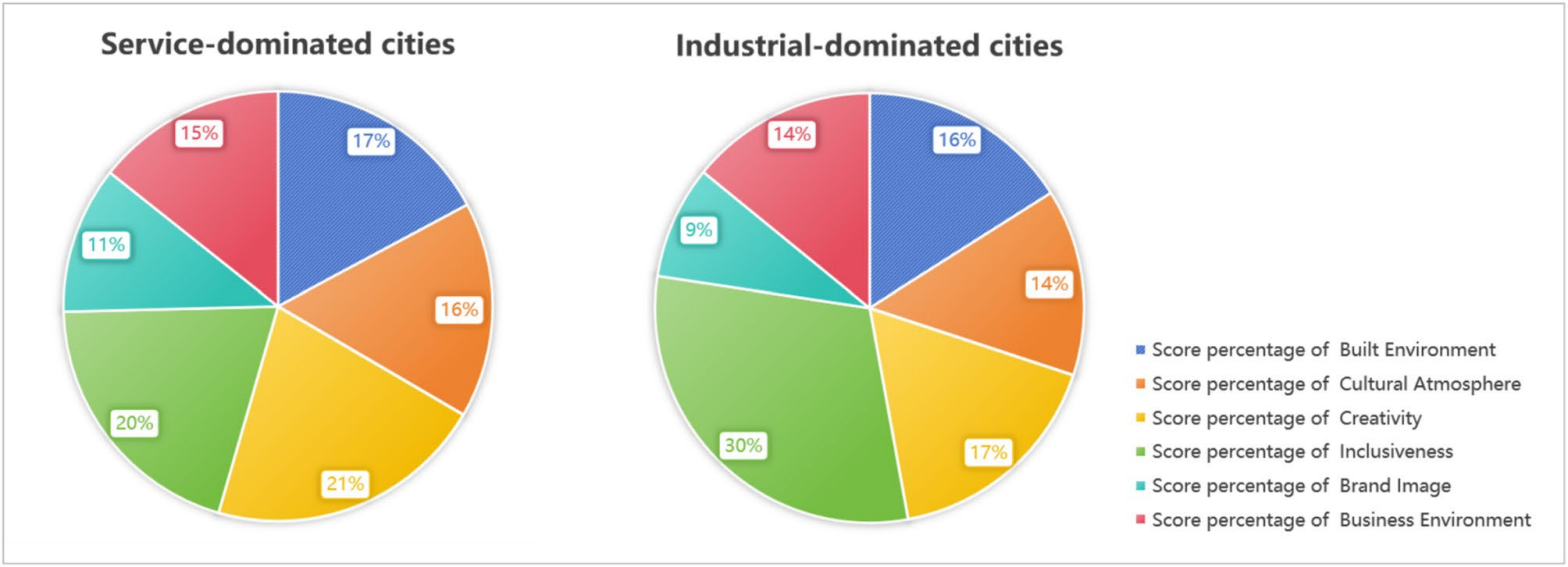
Comparison of neighborhood quality between service-dominated and industrial-dominated cities in China (Detailed data are in Appendix 4).

(6) Lastly, considering that each city has its own focus, either on industry or service, we further explored the difference in neighborhood quality of cities with different focuses. With reference to the statistical data in the *China Statistical Yearbook 2023*^9^, we defined cities whose service sector accounts for more than 50% of its GDP as service-dominated cities, and others as industrial-dominated cities. We summarized and compared the neighborhood quality scores of the 2 types of cities. It can be seen from Fig. 14 that: (1) Between the 2 types of cities, the neighborhood quality scores of service-dominated cities are more balanced and more in line with the characteristics of high-quality neighborhoods. (2) Similar to the heterogeneity analysis above, there is no significant difference in neighborhood quality for “hard” environments (built environment and business environment) between service-dominated and industrial-dominated cities. (3) Service-dominated cities have significantly higher creativity scores than industrial-dominated cities. Service-oriented cities tend to exhibit more balanced performance across different dimensions of neighborhood quality. This pattern aligns with studies on urban economic structure, which suggest that service-oriented economies are often associated with higher levels of urban amenities, diversity, and attractiveness^26,34^.

## Regression-based explanatory analysis of neighborhood quality

To further examine the relationship between neighborhood quality and socioeconomic conditions, this subsection conducts a regression-based explanatory analysis. The dependent variable is the neighborhood quality score obtained from the entropy-weighted evaluation model. Since most explanatory variables are available at the city level, they are assigned to all neighborhoods within the same city.

The explanatory variables include GDP per capita, population density, industrial structure (measured by the share of tertiary industry), city administrative level, and regional dummy variables. In addition, nighttime light intensity is included as a proxy for local development intensity. GDP per capita and population density are log-transformed to reduce skewness. The regression model is estimated using ordinary least squares (OLS), with standard errors clustered at the city level to account for within-city correlation. It should be noted that this analysis is intended as an explanatory exercise rather than a causal identification strategy. The aim is to provide systematic evidence on the socioeconomic correlates of neighborhood quality and to complement the descriptive heterogeneity analysis presented above.

To examine the relationship between neighborhood quality and city-level socioeconomic factors, the following regression model is specified:

1 $${Q}_{i}=\alpha +{\beta}_{1}ln\_{GDP}_{c}+{\beta}_{2}ln\_{Pop}_{c}+{\beta}_{3}TertiaryShar{e}_{c}+{\beta}_{4}CoreCit{y}_{c}+{\beta}_{5}NightLigh{t}_{c}+\delta Regio{n}_{c}+{e}_{i}$$

where ${Q}_{i}$ denotes the neighborhood quality score for neighborhood i. c is the city where the block i is located. $ln\_{GDP}_{c}$ represents the logarithm of GDP per capita in city c, capturing the level of economic development. $ln\_{Pop}_{c}$ denotes the logarithm of the resident population in city c, reflecting population agglomeration. $TertiaryShar{e}_{c}$ refers to the share of the tertiary industry in city c, measuring the structure of the local economy. $CoreCit{y}_{c}$ is a dummy variable indicating city administrative level, which takes the value of 1 if the city is classified as a core city and 0 otherwise. $NightLigh{t}_{c}$ represents nighttime light intensity in city c, serving as a proxy for local economic activity and development intensity. $Regio{n}_{c}$ is a regional dummy variable, which equals 1 if the city is located in eastern China and 0 otherwise. ${e}_{i}$ is the error term. All regressions are estimated using ordinary least squares (OLS), with standard errors clustered at the city level.

Table 5 reports the results of the regression-based analysis. Three specifications are estimated to examine the relationship between neighborhood quality and socioeconomic factors.

**Table 5:** Socioeconomic correlates of neighborhood quality.

Variables	(1)	(2)	(3)
	Modelmain1	Modelmain2	Modelmain3
	Q	Q	Q
ln(GDPc)	0.00219**	0.00169*	0.00276***
	(0.000920)	(0.000880)	(0.000975)
ln(Popc)	-0.00202*	-0.00119	-0.000560
	(0.00107)	(0.00107)	(0.00111)
TertiaryShare		0.000253***	0.000231***
		(4.77e-05)	(4.88e-05)
CoreCity		0.000153*	0.000665**
		(0.00609)	(0.00913)
NightLighti​			8.75e-05**
			(4.19e-05)
Region			0.00170*
			(0.000879)
Constant	0.0990***	0.103***	0.111***
	(0.00792)	(0.00905)	(0.00954)
City	Yes	Yes	Yes
Observations	5,829	5,829	5,829
R-squared	0.803	0.769	0.711

Standard errors in parentheses ***p < 0.01, **p < 0.05, *p < 0.1

In Model (1), which includes only basic economic variables, GDP per capita is positively and significantly associated with neighborhood quality, indicating that neighborhoods in more economically developed cities tend to exhibit higher overall quality. Population density shows a negative coefficient and is weakly significant, suggesting that higher density may be associated with certain pressures on neighborhood environments, although the magnitude of this effect is relatively small. Model (2) introduces variables related to industrial structure and city administrative level. The coefficient of GDP per capita remains positive and statistically significant, confirming the robustness of its association with neighborhood quality. The share of tertiary industry is positively and strongly significant, suggesting that service-oriented economic structures are closely linked to higher-quality neighborhood environments. In addition, core cities tend to exhibit higher neighborhood quality, reflecting their advantages in resource allocation and public service provision. The effect of population density becomes statistically insignificant in this specification, indicating that its influence may be mediated by other socioeconomic factors. Model (3) further incorporates nighttime light intensity and regional controls. The coefficient of GDP per capita remains positive and highly significant. Nighttime light intensity is also positively associated with neighborhood quality, suggesting that areas with higher levels of economic activity and development intensity tend to exhibit better neighborhood conditions. Regional differences remain statistically significant, indicating that spatial disparities persist even after controlling for key socioeconomic variables.

Overall, the regression results are broadly consistent with the descriptive findings presented above. Economic development, service-oriented industrial structure, and city hierarchy are positively associated with neighborhood quality, while the effect of population density appears to be more complex and context-dependent. Given the cross-sectional nature of the data and the use of city-level explanatory variables, the explanatory power of the model is limited in capturing all sources of variation, which is consistent with similar neighborhood-level studies. These results should therefore be interpreted as statistical associations rather than causal relationships.

To further explore the heterogeneous effects of socioeconomic factors on different dimensions of neighborhood quality, we re-estimate the regression model using soft-environment and hard-environment scores as dependent variables. The results are reported in Table 6.

**Table 6:** Heterogeneous effects on soft and hard environment dimensions.

Variables	(1)	(2)
	Modelmain1	Modelmain2
	soft-environment	hard-environment
ln(GDPc)	0.00168***	0.000666**
	(0.000471)	(0.000260)
ln(Popc)	0.000121	5.51e-05
	(0.000535)	(0.000295)
TertiaryShare	5.20e-05**	4.15e-05***
	(2.35e-05)	(1.30e-05)
CoreCity	0.000804*	0.000591*
	(0.000458)	(0.000302)
NightLighti	5.68e-05***	4.20e-05**
	(2.02e-05)	(3.12e-05)
Region	0.00115***	0.000663***
	(0.000424)	(0.000234)
Constant	0.0666***	0.0150***
	(0.00461)	(0.00254)
City	Yes	Yes
Observations	5,829	5,829
R-squared	0.691	0.731

Standard errors in parentheses ***p < 0.01, **p < 0.05, *p < 0.1.

Overall, the model exhibits relatively high explanatory power for both dimensions, suggesting that the selected socioeconomic variables are closely related to variations in neighborhood quality.

GDP per capita is positively and significantly associated with both soft and hard environment scores, indicating that economic development contributes to improvements in multiple aspects of neighborhood quality. Notably, the coefficient is larger for the soft environment, suggesting that economic development may play a more prominent role in enhancing cultural, social, and perceptual dimensions. Population density does not show a statistically significant effect in either specification, indicating that its influence on neighborhood quality is not straightforward and may depend on more complex urban dynamics. The share of tertiary industry is positively associated with both dimensions, with strong statistical significance. This result suggests that service-oriented economic structures contribute not only to physical improvements but also to the enhancement of soft-environment characteristics, such as cultural vitality and inclusiveness. Core cities exhibit higher scores in both dimensions, reflecting their advantages in resource concentration, infrastructure provision, and public service delivery. The magnitude of the coefficients is comparable across the two models, indicating that city hierarchy affects both material and non-material aspects of neighborhood quality. Nighttime light intensity is also positively and significantly associated with both dimensions, suggesting that higher levels of local development intensity and economic activity are linked to better neighborhood environments. Regional differences remain significant, indicating that spatial disparities persist even after controlling for key socioeconomic factors.

Taken together, these results suggest that socioeconomic development influences both the hard and soft dimensions of neighborhood quality, with somewhat stronger effects observed for the soft environment. These findings further support the patterns identified in the descriptive heterogeneity analysis and are consistent with the quality-of-place literature, which emphasizes the increasing importance of soft and experiential factors in shaping urban attractiveness^11,52^. As with the main specification, these results should be interpreted as statistical associations rather than causal relationships.

## Research conclusions and policy recommendations

This study constructs a comprehensive evaluation indicator system for neighborhood quality; by using street view images and big data technologies, this study has obtained microscopic sub-districts data in China; and then, the entropy weight method is used to assess the quality of 5829 major neighborhoods in China. The evaluation results show that: (1) In China, the construction of “soft” environment is more important for boosting the neighborhood quality, and this trend maps the fact that people are paying more attention to such “soft” environment as urban inclusiveness, cultural diversity, and third space. (2) Most of the high-quality neighborhoods in China are located in economically developed regions, such as the Yangtze River Delta, Pearl River Delta, and Beijing-Tianjin-Hebei urban agglomerations, indicating that the improvement of neighborhood quality depends, to some extent, upon the economic level of a region. (3) By comparing the quality scores of high- and low-quality neighborhoods, it is found that high-quality neighborhoods are relatively balanced in all dimensions, without obvious deficiencies. In contrast, low-quality neighborhoods tend to showcase remarkable shortcomings in terms of creativity. (4) Lastly, this paper includes a heterogeneity analysis on the neighborhood quality of cities in different regions, at different administrative levels, and with different industrial focuses, which reveals that even though China’s construction efforts on “hard” environments of cities are pretty much the same, significant differences exist in those on “soft environment”. Besides, the neighborhood quality of eastern cities, core cities, and service-dominated cities are more balanced and more in line with the characteristics of high quality neighborhoods.

The empirical results reveal substantial heterogeneity in neighborhood quality across regions, city types, and neighborhood categories. Accordingly, policy responses should move beyond uniform approaches and adopt more differentiated strategies that correspond to specific development contexts.

 (1) Strategies for eastern core cities.

In eastern core cities, neighborhood quality is generally high, and the basic physical environment is relatively well developed. Under such conditions, further improvements are less likely to come from additional infrastructure investment. Instead, policy efforts should focus on enhancing the soft environment, particularly cultural atmosphere, inclusive governance, and the quality of creative ecosystems.

This includes improving the provision and accessibility of cultural facilities, fostering diverse and inclusive public spaces, and supporting the development of creative industries and knowledge-based activities. Strengthening these aspects may help sustain urban attractiveness and avoid stagnation in high-level development contexts.

 (2) Strategies for central and western cities.

For cities in central and western regions, neighborhood quality remains relatively uneven, with noticeable gaps in soft-environment dimensions. While continued improvement in infrastructure is still important, particular attention should be given to addressing deficiencies in cultural and creative resources.

Policies may focus on promoting the transformation of local cultural assets into accessible public resources, strengthening the spatial distribution of cultural facilities, and enhancing the role of educational and research institutions. Rather than replicating development models from more advanced regions, these cities may benefit from leveraging local characteristics to gradually build a more balanced neighborhood environment.

 (3) Strategies for lower-ranked neighborhoods.

For lower-ranked neighborhoods, the results indicate that improvements in individual dimensions do not necessarily translate into overall quality enhancement. In particular, relatively higher inclusiveness scores do not automatically imply balanced development.

Therefore, policy interventions should avoid focusing solely on improving individual indicators. Instead, a more integrated approach is needed, emphasizing the development of basic innovation capacity, public cultural provision, and economic support. This includes improving access to education and cultural services, supporting local economic activities, and strengthening connections to broader urban systems. Such measures may help promote more sustainable and structurally balanced neighborhood development.

 (4) Strategies for service-oriented and industrial cities.

Differences are also observed between service-oriented and industrial cities. Service-oriented cities tend to exhibit more balanced development across dimensions, particularly in soft-environment factors. In these cities, policy efforts may focus on further enhancing diversity, cultural vitality, and quality of public space.

In contrast, industrial cities often display relatively stronger performance in physical and economic dimensions but weaker performance in cultural and creative aspects. For these cities, it is important to avoid overemphasizing physical development while neglecting the cultural and social environment. Policies should therefore promote cultural infrastructure, improve public services, and foster more inclusive and diverse urban environments to support long-term transformation.

### Limitations and future research

Despite the contributions of this study, several limitations should be noted. The empirical analysis relies primarily on data around 2016, including POI data, nighttime light data, and related datasets. The results therefore reflect the spatial pattern of neighborhood quality at a particular stage of urban development in China. Given the rapid transformation of Chinese cities in recent years, caution is needed when extending these findings to more recent periods.

Street view images were obtained from an open platform, and their acquisition time is not fully consistent across locations. Although winter images in northern cities were excluded to reduce seasonal bias, some temporal heterogeneity inevitably remains. This issue has been widely discussed in street view-based urban studies, where differences in image acquisition time may introduce additional uncertainty (e.g^4^.,). In this study, such effects are likely to manifest as random variation rather than systematic bias, as the analysis is conducted at an aggregated neighborhood level with a large number of observations.

The POI data used in this study may also involve certain limitations. As with other forms of large-scale geospatial data, POI datasets may suffer from incompleteness, classification inconsistencies, or temporal lag^25^. These issues could affect the measurement of some dimensions, particularly those related to cultural atmosphere, inclusiveness, and business environment, where the identification of facilities plays a central role.

In addition, the semantic segmentation model is based on the Cityscapes dataset, which was developed using street scenes from European cities. Although the main categories used in this study—such as roads, buildings, vegetation, and sky—represent basic physical components that are relatively stable across urban contexts, transferring the model to Chinese cities may still introduce recognition bias. Similar challenges have been noted in studies applying computer vision models across different geographical contexts^30^. In this study, this issue is partly mitigated by focusing on visually distinguishable categories and by aggregating results at the neighborhood level, which reduces the influence of individual misclassification errors.

Future research could address these limitations in several ways. Incorporating multi-period data would allow for the analysis of temporal dynamics in neighborhood quality. Data quality could be improved through the integration and cross-validation of multiple sources. In addition, segmentation performance could be enhanced by fine-tuning models using annotated Chinese street view datasets. Finally, combining large-scale evaluation with regression or spatial econometric approaches would help to further explore the mechanisms underlying neighborhood quality.

## Supplementary Information

Supplementary Information.

## Data Availability

The datasets used and/or analysed during the current study available from the corresponding author on reasonable request.
