# Supplementary material for: Fine-grained evaluation of neighborhood quality in China using street view images and big data technologies
Source: Sci Rep. 2026 Jun 1;16:25049. doi: 10.1038/s41598-026-53650-w (PMC13462419; doi:10.1038/s41598-026-53650-w)
Supplement: Supplementary file 1 [file 41598_2026_53650_MOESM1_ESM.docx]

# Appendix 1

# (B1) Built Environment

The two indicators (urban aesthetics and urban environment) used by Florida to describe the urban appearance are collectively referred to as Built Environment. When selecting the indicators for assessing Built Environment, this study has, in addition to the building density and green environment indicators used by Florida, added the following indicators into the evaluation system for basic environment: the permeability of streetscape mentioned by Montgomery (2003) in assessing local comfort; and the traffic fluency mentioned by Ma et al. (2018) in assessing quality of place in China's cities. Finally, it is determined to evaluate Built Environment from four directions: Traffic fluency (C01), Building density (C02), Green visible index (C03), and permeability (C04). Good traffic fluency means higher motorization and connectivity, bringing good habitual experience to residents (Gehl, 2002). Higher building density reflects stronger efforts in urban construction and a higher degree of urban space utilization, and thus a higher urbanization level (Tang & Long, 2019). A higher green visible index increases users’ perceived safety in the space and also relieves people’s mental stress (Li et al., 2015). From a visual perspective, a street with higher permeability makes its users feel safer (Montgomery, 2003). (refer to Fig. 1).

## Built Environment

**Research subjects**

**Individual perceptions**

**Individual indicators**

**Comprehensive indicators**

**Roads**

**Vehicles**

**Buildings**

**Plants**

**Sky**

**Walls**

**Sense of space**

**Greenery degree**

**Comfort level**

**Congestion feeling**

**Traffic fluency (C1)**

**Density of buildings (C2)**

**Green visible index (C3)**

**Permeability of streetscape (C4)**

**Neighbourhood appearance features**

### Fig.1: Framework for Assessing built environment

(C01) Traffic Fluency. Traffic flow is an important indicator of a city's spatial accessibility. It is typically measured by using the physical dimensions of road interfaces, such as the separation between buildings or structures (walls, fences) on either side of the street, or a combination of secondary spatial scales, such as motorways, non-motorized lanes, and pavements (Li et al.,2019). This paper evaluates street patency at each street view collection point using the proportion of sidewalks, vehicle lanes, and the openness of both. The applicable formulas are as follows:

$C011_{\text{j}}=\sum_{\text{i}=1}^{NUM_{\text{j}}} R\text{oad}_{\text{ij}}$ (1)

$C012_{\text{j}}=\sum_{\text{i}=1}^{NUM_{\text{j}}} S\text{idewalk}_{\text{ij}}$ (2)

$C013_{\text{j}}=\sum_{\text{i}=1}^{NUM_{\text{j}}} \frac{Person_{\text{ij}}+rider_{\text{ij}}+bi\text{cycle}_{\text{ij}}}{R\text{osd}_{\text{ij}}}$ (3)

$C014_{\text{j}}=\sum_{\text{i}=1}^{NUM_{\text{j}}} \frac{C\text{ar}_{\text{ij}}+T\text{ruck}_{\text{ij}}+B\text{us}_{\text{ij}}+M\text{otorcycle}_{\text{ij}}}{S\text{idewalk}_{\text{ij}}}$ (4)

$C015_{\text{j}}=\sum_{\text{i}=1}^{NUM_{j}} T\text{raffic-light}_{\text{ij}}+T\text{raffic-sign}_{\text{ij}}$ (5)

The variables C011j, C012j, C013j, C014j and C015j represent the relative abundance of sidewalks, vehicle lanes, and open space in neighbourhood j, respectively. The number of street images gathered in neighbourhood J is denoted by Nj. Also included are the pixel proportions for the following in the i-th street view of neighbourhood j: motorised lanes, non-motorized lanes, sidewalks, pedestrians, cyclists, bicycles, automobiles, trucks, buses, and motorcycles. ${Traffic-light}_{\mathrm{ij}} \& {Traffic-sign}_{\mathrm{ij}}$ are the pixel proportions of traffic lights and signs in the i-th street view of City j, respectively.

(C02) Building Density. Building density reflects the ratio of open space to unit area as well as building coverage density. A higher building density indicates more intensive urban development and better use of urban space. This density is calculated as the ratio of building area to total area of a region, as determined by the recognition results of street view images.

$C021_{\text{j}}=\sum_{\text{i}=1}^{NUM_{j}} B\text{uilding}_{\text{ij}}$ (6)

Where C021j represents the development intensity of neighbourhood j, and Buildingij represents the pixel proportion of buildings in the i-th street view image of neighbourhood j.

(C03) Green Visible Index (GVI). This metric provides information about the user's spatial experience while objectively assessing the quality of an urban public green space. Scholars usually measure GVI by the proportion of green elements within the visual field (Long and Liu,2017). In this study, the pixel proportion of identified plant elements in street view images is used to compute the neighbourhood's green visibility rate. The calculation formula is provided below.

$C031_{\text{j}}=\sum_{\text{i}=1}^{NUM_{j}} V\text{egetation}_{\text{ij}}$ (7)

Where C031j represents the GVI of neighbourhood j, and Vegetationij represents the pixel proportion of plant elements in the i-th street view image of neighbourhood j.

(C04) Permeability of streetscape. Permeability of streetscape is primarily measured by the proportion of walls and sky in street views (Li et al.,2019). The wall proportion, which represents the length of the enclosing walls on both sides of the street in relation to the total street length, indicates street interface continuity. This study quantifies it by adding up the image elements (buildings, walls, and fences) along pedestrian paths on both sides and dividing by twice the length of the street. The formula is expressed as follows.

$C041_{j}=\sum_{\text{i}=1}^{NUM_{j}} \frac{B\text{uilding}_{\text{ij}}+W\text{all}_{\text{ij}}+F\text{ence}_{\text{ij}}}{2\times ALL_{\text{ij}}}$ (8)

Conversely, sky proportion, which represents pedestrian spatial enclosure, correlates inversely with permeability; higher permeability implies a better sightline perception. This study calculates it as the proportion of sky area in the total street view image area. The formula goes as follows:

$C042_{\text{j}}=\sum_{\text{i}=1}^{NUM_{j}} \frac{S\text{ky}_{\text{ij}}}{ALL_{\text{ij}}}$ (9)

Here C041j denotes the street wall proportion and C042j denotes the sky proportion in neighbourhood j. The terms Wallij, Fenceij, and Skyij refer to the pixel proportions of wall, railing, and sky elements in the i-th street view of neighbourhood j, respectively, while ALLij refers to all pixels in the same street view (value is 1).

# (B2) Cultural Atmosphere

The urban vibrancy and culture mentioned by Florida are collectively referred to as the cultural atmosphere. When selecting the indicators for assessing the cultural atmosphere, Chinese scholars usually take a dual material and spiritual approach to quantify cultural atmosphere. For example, Chen et al. (2017) maintains that cultural consumption, cultural facilities and historical depth deliver important influences on a city's cultural ambience, with an emphasis on user experience, while advocating a three-part assessment of cultural ambience in terms of material, spirit, and institution. This study adopts the Chinese academic perspective to evaluate the neighborhood cultural environment in two dimensions: material and spirit. Based on Chen et al.'s (2017) ^24^approach, this study adopts cultural facilities (C05) as an indicator to assess the material aspect of neighborhood cultural atmosphere. The spiritual dimension is assessed by the perception of public environment, which includes historical profundity (C06) and natural resources (C07). The abundance of cultural facilities secures a dense cultural atmosphere, serves as an important material carrier that showcases and communicates urban culture, lays a foundation for the public to participate in cultural activities, and reflects the government’s level of public cultural service. Historical profundity carries the rich culture of a city, witnesses the important events in its history, and serves as one of the carriers of cultural atmosphere of the city and a window through which the public looks to the history. And natural resources provide a space for the public’s tour or scientific and cultural activities. Therefore, more abundant cultural facilities, historical profundity, and natural resources in a neighborhood are associated with stronger cultural atmosphere and thus higher neighborhood quality. Table 2 details the data acquisition methods.

**Neighbourhood cultural atmosphere**

**Cultural facilities (C5)**

**Historical profundity (C6)**

**Natural resources (C7)**

Material aspect

Spiritual aspect

Fig.2. Framework for Evaluating Cultural Atmosphere in Neighbourhoods

(C05) Cultural Facilities. Cultural facilities in a neighbourhood are essential for promoting public cultural activities. Their abundance reflects a city's vibrant cultural atmosphere and serves as an important material medium for transmitting urban culture. These facilities influence societal values, concepts, preferences, and behaviours, demonstrating the city's level of civilization and development. This study quantifies cultural facilities by counting the number of museums, public libraries, and theatres in the neighbourhood.

(C06) Historical Profundity. The historical depth of a neighbourhood, as a repository of a city's cultural heritage, has a significant impact on the cultural environment. It symbolises the neighbourhood's developmental history, embodying distinct spiritual values, historical memories, and creative essence. This study assesses historical profundity by counting units of contemporary cultural significance and historical preservation in the neighbourhood.

(C07) Natural Resources. Natural resources in a neighbourhood are those that have been modified and used to provide material resources and space for human activities. Scenic areas, for example, provide valuable spaces for daily sightseeing, exploration, learning, and cultural exchanges. Similarly, parks play an equivalent role in residents' daily lives. This paper evaluates natural resources by counting National AAAAA Class Scenic Spot, National AAAA Class Scenic Spot, National AAA Class Scenic Spot, National AA Class Scenic Spot, National A Class Scenic Spot and green parks in the neighbourhood.

# (B3) Creativity

The technology innovation, talent and creativity (Bohemian) mentioned in Florida in assessing the quality of place are all reflecting a city's creativity. Hence, this study refers to these dimensions collectively as Creativity. In reference to Trip's (2007) approach, the number of creative institutions is used to describe the creativity of a community. So the indicators of university (C08), national key laboratory (C09), scientific research institution (C10) are selected to assess the Creativity of a community. All the 3 types of institutions serve as the main producers and providers of talent and technology. Scientific and educational development is conceived in universities which nurture talents for society, communicate culture, conduct scientific research, and convert the results of scientific research, etc. National key laboratories conduct basic research and applied basic research in frontier science, basic science, and engineering science. Scientific research institutions, as an integral part of science and education innovation, primarily work on research in basic disciplines, product R&D, and conversion of scientific and technological results. Therefore, the more universities, national key laboratories and scientific research institutions in a neighborhood, the stronger its creativity and the higher its neighborhood quality.

(C08) University to research platforms: Universities are important research platforms, and their number has a direct impact on a region's scientific and educational standards. This study uses the number of colleges and universities, categorising them as "985", "211", and other types in accordance with China Education Online Network 3 classification standards, to assess the district's college resource level.

(C09) National Key Laboratories. They are fundamental to national science and technology innovation bases and are critical to original innovation capabilities. This study quantifies the district's key laboratory resources by counting the number of state-key laboratories housed within businesses, those established in collaboration with provinces and ministries, and those affiliated with universities.

(C10) Scientific Research Institutions. They, which include various research institutes, are quantified in this study by the number of Chinese Academy of Sciences institutes in the neighbourhood, indicating the neighbourhood's level of scientific research institute resources.

# (B4) Inclusiveness

Florida's assessment of quality of place fully reflects his understanding of social values: he made evaluation in terms of urban tolerance and openness. When describing local comfort, Clark et al. (2002) also underscores the importance of social values, such as friendliness, tolerance, adventure, and individualism. In this study, these elements used to describe the social values of cities are collectively referred to as inclusiveness. And the three concepts of inclusive urban development as defined by Catherine and Thomas (2019) are adopted, who maintains that inclusiveness of neighborhoods is embodied in calling for equal development opportunities and shared development benefits; Moreover, planning shall be made from the perspectives of the disadvantaged groups, in order to benefit a wider range of users, while delivering the goal of helping the disadvantaged groups to integrate into the mainstream social groups. From an inclusiveness perspective, neighborhood space should reflect these values. Therefore, the three criteria for inclusive neighborhood space are determined: fairness and justice (C11), respect for diversity (C12), and people oriented (C13).And a neighborhood that features fairness and justice, respect for diversity, and people oriented tends to have higher quality.

### Fig.3. Neighbourhood inclusiveness assessment framework

(C11) Fairness and Justice. According to Cao and Zhuo (2017), an inclusive neighbourhood should ensure that all residents have equal access to public space resources. To achieve "fairness and justice," space allocation for pedestrians and vehicles must be balanced during neighbourhood construction. The frequent conflict between pedestrian and vehicular spaces emphasises the issue of resource inequality. Expanding roads in modern car-centric urban transportation systems may reduce traffic congestion, but it often comes at the expense of sidewalk space, disrupting pedestrian continuity. Furthermore, improving road infrastructure and traffic sign clarity can moderately increase local resource utilisation. This paper assesses the pedestrian-vehicle space balance using the pixel proportion of sidewalks, roadways, and traffic infrastructure identified in street view images. The completeness of a neighbourhood's traffic infrastructure is measured by the number of pixels on traffic signals and signs. The following formulas are used:

$C111_{\text{j}}=\sum_{\text{i}=1}^{NUM_{j}} \frac{S\text{idewalk}_{\text{ij}}}{R\text{oad}_{\text{ij}}}$ (10)

Where, C111j represents the pedestrian-vehicle balance in neighbourhood j。

In addition, we use the percentage of the disadvantaged groups population (age 65+ and under 15) in each neighborhood from the national sixth and seventh population census for the quantification of fairness and justice.The following formulas are used:

$C112_{\text{j}}=\frac{WEAK\_POP_{\text{j}}}{NUM\_POP_{\text{j}}}$ (11)

C112j denotes the percentage of the disadvantaged group (age 65+ and under 15) population in neighborhood j. WEAK_POPj represents the number of disadvantaged populations in neighborhood j, and NUM_POPj represents the total population in neighborhood j.

The number of traffic facility service units (bus stops, subway stations, long-distance bus stations, gas stations, toll booths, bridges, and service stations) calculated from POI data is used to evaluate the neighbourhood's traffic infrastructure.

(C12) Respect for Differences.A fully functional neighbourhood with comprehensive commercial, recreational, and residential facilities can meet a variety of individual needs. 同时还要包容外来人群。This study analyses commercial spatial patterns in sub-districts using POI data to assess neighbourhood functional diversity. Ten urban functions that influence neighbourhood quality are identified, and statistical scores are assigned based on China's Urban Construction Land Classification and Li's (2019) methodology (scoring standards detailed in Table 1). The functional diversity index of neighbourhoods is then computed as follows:

$C121_{\text{j}}=\frac{\text{A}1_{\text{j}}\times6+\text{A}2_{\text{j}}\times6+\text{A}3_{\text{j}}\times5+A4_{\text{j}}\times5+\text{A}5_{\text{j}}\times10+\text{A}6_{\text{j}}\times10+A7_{\text{j}}\times5+\text{A}8_{\text{j}}\times6+A9_{\text{j}}\times8+\text{A}10_{\text{j}}\times5}{NUM\_POI_{\text{j}}}$ (12)

### Table 1 . Statistical Scoring of Functional Units

| **ID** | **Site** | **scoring** | **ID** | **Site** | **scoring** |
| --- | --- | --- | --- | --- | --- |
| A1 | Recreation | 6 | A6 | Market | 10 |
| A2 | Medical | 6 | A7 | Finance | 5 |
| A3 | Educational | 5 | A8 | Media | 6 |
| A4 | Service | 5 | A9 | Hotel | 8 |
| A5 | Catering | 10 | A10 | Exercise | 5 |

Where, C121j denotes the functional diversity index for neighbourhood j, with higher values indicating greater functionality. A1j - A10j are the number of leisure and entertainment, medical, educational, and training, life service, food and catering, shopping, financial, cultural media, hotel accommodation, and sports and fitness units in neighbourhood j that was extracted from POI data; NUM_POIj is the total count of functional units in neighbourhood j obtained from POI data.

In addition, this study uses the percentage of the outlanders' population in each neighborhood from the Sixth and Seventh Census data to measure the neighborhood's tolerance of non-locals.

$C122_{\text{j}}=\frac{OUT\_POP_{\text{j}}}{NUM\_POP_{\text{j}}}$ (13)

C122j denotes the percentage of outlanders' population in neighborhood j, where OUT_POPj represents the number of outlanders in neighborhood j.

(C13) People Oriented. This concept emphasises putting people first, from neighbourhood planning and design to value orientation, ensuring that both physiological and psychological needs are thoroughly addressed (Liang,2017). Furthermore, neighbourhood designs should consider not only the physical needs of residents, but also their dynamic lifestyles. Inclusive neighbourhoods must therefore take into account both human physiological and psychological levels. While creating functional material spaces, they should also consider the cultural, social, and emotional needs of various user groups.

This paper evaluates the neighbourhood's material completeness and psychological satisfaction across two domains. Material completeness is determined by the number of hospitals(Level-III hospitals, level-II hospitals, level-I hospitals, other hospitals）, living service facilities, and scientific, educational, and cultural service institutions in the area. Neighbourhood hospitals include all class-A hospitals in the area. Living service institutions are classified into 14 types, including post offices, logistics company ticket offices, laundries, photo and graphic printing shops, real estate agencies, public utilities, maintenance points, housekeeping services, funeral services, lottery sales points, pet services, newsstands, and public toilets. There are ten types of scientific, educational, and cultural service institutions: middle schools, primary schools, kindergartens, adult education centres, parent-child education facilities, special education schools, international study agencies, training institutions, libraries, and science and technology museums.

# (B5) Brand Image

Florida (2002) believes that the value of “impact” to city users is reflected in the improvement of urban image and residents’ confidence, and he takes public attention as a criterion for measuring the impact of a city in a research about the measurement of US cities. Qi et al. (2009) consider it a measurement of “breadth”, i.e. the number of public that is familiar with the city. Aside from that, some scholars hold that a city’s impact should also reflect “depth”, i.e. the public’s degree of understanding of entities (Xia and He, 2009). This paper therefore expands the “impact” mentioned by Florida to incorporate the “depth” of the public’s understanding of urban neighborhoods, and refers to such measurement indicator that considers both “breadth” and “depth” as brand image. In essence, the breadth (C14) for brand image means how many people can identify the entities and the depth (C15) means how well they know those entities. A neighborhood with a quality brand image can not only drive the economic development of the city, but also improve the image of the whole city. Brand image is an important way to elevate neighborhood quality., using data acquisition methods described in Table 4.

(C14) Breadth. This study utilises Zhao's (2023) method to assess breadth, employing as a proxy for public awareness of the frequency of neighbourhood-related keywords in prominent Chinese internet search engines such as Baidu, Google, and 360. This methodology is consistent with the approach utilised by Qian and He (2010) to examine the impact of 5A-level tourist attractions on the brand image of a city.

(C15) Depth. Gong et al.(2018) provides the methodology for the depth measurement, which evaluates public perceptions of organisations according to the recognition or honours they have obtained. By comparing lists of renowned Chinese historical and cultural villages, towns, and streets, this article determines whether a neighbourhood has been bestowed with national honours, thereby quantifying the depth of its brand image.

# (B6) Business Environment

The indicators of urban diversity and recreational facilities mentioned by Florida are used to describe the concept of "what is available, i.e., the abundance of facilities". However, there is a large overlap between these two indicators. For example, when describing local comfort, Albouy and Stuart (2020) collectively refers to these elements as urban services; Brueckne et al. (1999) refers to them as modern facilities; and Trip collectively refers to these two indicators as specific facilities. In China, however, either urban services or leisure places fall into the category of business development (Chen, 2015) . Therefore, this study categorizes urban diversity and recreational facilities as business environment, and evaluates it in three directions: commercial vitality (C16), business diversity (C17), and market size (C18). Commercial vitality represents the level of commercial development in a neighborhood; business diversity reflects business formats and business modes (Wade, 1997); and market size shows the business volume. In this regard, higher commercial vitality, more diversified businesses, and larger market size of a neighborhood are correlated to a better business environment and thus higher neighborhood quality.

(C16) Commercial Vitality. Commercial vitality is the foundational element of the business environment. This study uses NOAA's global 2016 annual NPP-VIIRS night light remote sensing data to measure neighbourhood commercial vitality. Xi et al.(2013) found a significant relationship between global night light data and GDP, demonstrating its usefulness in assessing regional economic development. This data, obtained through satellite sensors, transcends price levels, administrative divisions, and geographic scales.

(C17) Business Diversity. Business diversity refers to the presence and proximity of various commercial entities within a neighbourhood, indicating the variety of commercial types. The study uses POI data to determine the diversity of commercial functions in the research neighbourhood, and the number of street images collected for each neighbourhood corresponds to its length. Li's (2019) method is used to calculate the functional diversity of each neighbourhood, as shown in the formula below:

$C171_{\text{j}}=\frac{NUM\_POI_{\text{j}}}{NUM_{\text{j}}}$ (14)

Where, C171j denotes the functional diversity of neighbourhood j, NUM_POIj represents the total number of commercial functions in neighbourhood j, and NUMj denotes the number of street scenes collected for each neighbourhood, which is used to represent its length.

(C18) Market Size. The availability of goods and services in a neighbourhood indicates market size, which is an important factor in determining a commercial area's supply capacity. A larger market size is typically associated with greater economic benefits, attracting more businesses and creating a positive cycle that improves the neighbourhood's commercial atmosphere. In this study, the number of merchants identified in POI data is used to estimate a neighbourhood's market size.

# Appendix 2

# In order to facilitate the follow-up research, it is necessary to match all kinds of data to form a block-level research data set. The specific matching steps are as follows:

The first step is to determine the research sample. This paper introduces the method of invoking the API of Baidu Street View through Python software to collect more than 7.8 million Baidu Street View images from 5829 main township streets in 232 cities in China. Since the deep learning of Baidu Street View is the core content of this paper's street quality assessment, this paper takes the 5829 main townships, towns and streets that can be collected from street view pictures as the research object of this paper. All subsequent data collection will also be carried out around the 5,829 townships, towns and streets.

The second step is to unify the sample names. The data obtained in this paper are mainly from Baidu Street View images of open network data, city point of interest (POI) data, NPP-VIIRS night light remote sensing data and the conversion of various file lists. Because of the different ways of acquiring blocks, different kinds of data sets have different ways of naming blocks. For example, the block name obtained through the deep learning of Baidu Street View map comes from the naming content of the collected image, which contains the name of the block to which the collection point belongs. However, if the administrative division of the block corresponding to the collection point belongs to the "street" level, then the name will only be displayed as "so-and-so street". The names of the blocks in the data converted from POI data, lighting data and reverse geocoding tools are relatively complete. Therefore, we carried out supplementary processing on the block names whose names end with the word "street" in the samples obtained by the deep learning of Baidu Street View, and uniformly modified them to "street". In addition, considering that different cities may have blocks with the same name, we adopted the format of "so-and-so city" + "so-and-so town/street/township" as the unified naming method for the research samples.

Since in the process of mining street view images, this paper is based on the collection points generated by different prefecture-level cities , it is only necessary to match the street view images collected at the collection points with the prefecture-level cities to which the collection points belong, and then complete the deep learning of Baidu Street View images to obtain sample name modification. The data converted by POI data, lighting data and reverse geocoding tools not only includes the information of the block to which each sample belongs, but also the name of the city to which each sample belongs, so combining the two can complete the name unification of the sample.

The third step is the matching of block data. The stata tool was used to match the POI data with unified name, light data and reverse geocoding tool with the unified block name as the keyword and 5829 sample blocks. All kinds of objects belonging to the research sample can be obtained by removing the part that fails to match. In the follow-up study, it is only necessary to set the screening conditions according to the actual needs, and then count the blocks according to the block names, so that various data contents of 5829 blocks in the study sample can be obtained. All the data are collected at the block level according to the detailed address information in the data, and the detailed data of 5829 blocks studied in this paper are selected as the basic data for subsequent indicator evaluation. The final descriptive statistics of the tertiary indicators are shown in Table 3.

Table 3 Descriptive statistics of tertiary indicators

| Sample name | Number of samples | | Mean value | Standard deviation | Minimum value | Maximum value |
| --- | --- | --- | --- | --- | --- | --- |
| C011 | | 5829 | 197.697 | 400.562 | 0 | 6080.92 |
| C012 | | 5829 | 30.471 | 54.488 | 0 | 948.403 |
| C013 | | 5829 | 0.278 | 0.491 | 0 | 28.596 |
| C014 | | 5829 | 1.411 | 0.89 | 0.004 | 24.284 |
| C015 | | 5829 | 6.809 | 10.696 | 0 | 246.467 |
| C021 | | 5829 | 196.876 | 305.667 | 0.001 | 11964.775 |
| C031 | | 5829 | 300.641 | 413.945 | 0.007 | 4838.09 |
| C041 | | 5829 | 113.248 | 170.704 | 0.001 | 6218.568 |
| C042 | | 5829 | 338.715 | 490.605 | 0.005 | 6372.713 |
| C051 | | 5829 | 0.378 | 1.286 | 0 | 34 |
| C052 | | 5829 | 0.85 | 1.889 | 0 | 26 |
| C053 | | 5829 | 0.009 | 0.104 | 0 | 2 |
| C061 | | 5829 | 0.028 | 0.191 | 0 | 4 |
| C062 | | 5829 | 0.105 | 0.437 | 0 | 10 |
| C071 | | 5829 | 0.115 | 0.098 | 0 | 4 |
| C072 | | 5829 | 0.244 | 1.526 | 0 | 21 |
| C073 | | 5829 | 0.298 | 1.912 | 0 | 27 |
| C074 | | 5829 | 0.325 | 1.025 | 0 | 14 |
| C075 | | 5829 | 0.812 | 2.123 | 0 | 31 |
| C076 | | 5829 | 7.75 | 10.152 | 0 | 65 |
| C081 | | 5829 | 0.004 | 0.069 | 0 | 2 |
| C082 | | 5829 | 0.009 | 0.11 | 0 | 4 |
| C083 | | 5829 | 0.268 | 0.702 | 0 | 9 |
| C091 | | 5829 | 0.005 | 0.072 | 0 | 1 |
| C092 | | 5829 | 0.01 | 0.102 | 0 | 2 |
| C093 | | 5829 | 0.028 | 0.187 | 0 | 3 |
| C101 | | 5829 | 0.01 | 0.103 | 0 | 2 |
| C111 | | 5829 | 0.357 | 0.371 | 0.017 | 11.127 |
| C112 | | 5829 | 0.214 | 0.315 | 0.124 | 0.429 |
| C113 | | 5829 | 9.015 | 11.272 | 3 | 261.9 |
| C121 | | 5829 | 5.897 | 1.404 | 0 | 10 |
| C122 | | 5829 | 0.365 | 0.255 | 0.187 | 0.319 |
| C131 | | 5829 | 0.863 | 1.456 | 0 | 13 |
| C132 | | 5829 | 2.551 | 8.253 | 0 | 85 |
| C133 | | 5829 | 15.636 | 24.552 | 0 | 111 |
| C134 | | 5829 | 69.351 | 124.356 | 0 | 855 |
| C135 | | 5829 | 260.066 | 330.476 | 0 | 3905 |
| C136 | | 5829 | 77.071 | 96.043 | 0 | 1348 |
| C141 | | 5829 | 1252573.9 | 6596463 | 256 | 109200000 |
| C142 | | 5829 | 1338629.6 | 6968004.2 | 245 | 107500000 |
| C143 | | 5829 | 1331665.7 | 6939294.6 | 214 | 108700000 |
| C151 | | 5829 | 0.08 | 0.272 | 0 | 1 |
| C161 | | 5829 | 10.032 | 11.192 | 0.009 | 70.351 |
| C171 | | 5829 | 23.916 | 311.733 | 0 | 15075 |
| C181 | | 5829 | 2282.747 | 2875.607 | 1 | 37573 |

# Appendix 3: Detailed Steps of using entropy weight method to measure neighborhood quality

The evaluation of neighborhood quality using entropy weight method is divided into seven steps: data acquisition and collation, basic data standardization, indicator gravity measurement, information entropy measurement, difference coefficient measurement, indicator weight measurement, weighted summation. The details are as follows:

**Data collection and organization**

**Standardization of basic data**

**Calculation of indicator weights**

**Calculation of information entropy**

**Calculation of variation coefficient**

**Calculation of index weights**

**Weighted summation**

### FIG. 10 Summary diagram of evaluation steps by entropy weight method

Step 1: Data acquisition and collation. The indicators in this paper are derived from Baidu Street View image, urban point of interest (POI) data, NPP-VIIRS night light remote sensing data and urban spatial geographic information system (GIS). Matrix arrangement of all data, the number of selected blocks is represented by n, the number of indicators of each block is represented by m, all data is represented by an n*m matrix A, n is the number of recording rows of blocks, m is the number of recording columns of indicators, Xij in the matrix represents the value of the JTH indicator of the i block (i= 1,2... , n; j= 1,2,... m). Due to the large number of samples, we selected 10 groups of samples (the neighborhoods where famous universities such as Peking University and Tsinghua University are located, see Table4 for details of the calculation process.

$A=\left(\begin{matrix} X_{11} & ... & X_{1m} \\ ... & ... & ... \\ X_{n1} & ... & X_{nm} \end{matrix}\right)_{\text{n}\times\text{m}}$ （14）

### Table 4 shows the list of blocks during the calculation process

| **ID** | **Name of neighborhood** | **985-project universities in the neighborhoods** |
| --- | --- | --- |
| 1 | Qinghuayuan Subdistrict, Beijing | Peking University |
| 2 | Dongsheng Town, Beijing | Tsinghua University |
| 3 | Zhongguancun Subdistrict, Beijing | Renmin University and Beijing Institute of Technology |
| 4 | Wujiaochang Subdistrict, Shanghai | Fudan University |
| 5 | Xujiahui Subdistrict, Shanghai | Shanghai Jiao Tong University |
| 6 | Sipinglu Subdistrict, Shanghai | Tongji University |
| 7 | Cuiyuan Subdistrict, Hangzhou | Zhejiang University |
| 8 | Chigang Subdistrict, Guangzhou | Sun Yat-sen University |
| 9 | Yulin Subdistrict, Chengdu | Sichuan University |
| 10 | East Lake Scenic Area Subdistrict, Wuhan | Wuhan University |

Step 2: Standardized processing of basic data. Due to the different acquisition methods and reference evaluation methods of various indicators, the units of measurement of various indicators are not uniform. Therefore, before the measurement of indicators, it is necessary to carry out data standardization processing that the absolute value of each indicator is converted into relative value, so as to solve the homogenization problem of various heterogeneous index values. The specific algorithm is as follows:

$X_{\mathrm{ij}}^{’}=\frac{X_{\mathrm{ij}}-min（X_{1j}，...X_{\mathrm{nj}}）}{max（X_{1j}，...X_{\mathrm{nj}}）-min（X_{1j}，...X_{\mathrm{nj}}）}$	（15）

Xij 'is the data after standardized processing. A' is the data matrix after normalization, and the expression is as follows:

$A^{'}=\left(\begin{matrix} X^{'}_{11} & ... & X^{'}_{1m} \\ ... & ... & ... \\ X^{'}_{n1} & ... & X^{'}_{nm} \end{matrix}\right)_{\text{n}\times\text{m}}$ （16）

Table 5 shows the detailed presentation of basic data after standardized processing of selected samples.

### Table 5 shows the standardization of three levels of selected samples in detail**^[1]^**

| **ID** | **C′011** | **C′012** | **C′13** | **C′014** | **C′015** | **C′021** | **C′031** | **C′041** | **C′042** |
| --- | --- | --- | --- | --- | --- | --- | --- | --- | --- |
| 1 | 0.0019 | 0.0091 | 0.0348 | 0.0907 | 0.0381 | 0.0253 | 0.0556 | 0.0263 | 0.0332 |
| 2 | 0.0002 | 0.0002 | 0.0001 | 0.0011 | 0.0001 | 0.0001 | 0.0008 | 0.0001 | 0.0006 |
| 3 | 0.0028 | 0.0097 | 0.0181 | 0.0512 | 0.0352 | 0.0168 | 0.0506 | 0.0180 | 0.0495 |
| 4 | 0.0312 | 0.0305 | 0.0006 | 0.0345 | 0.0063 | 0.0043 | 0.0556 | 0.0059 | 0.0301 |
| 5 | 0.0115 | 0.0200 | 0.0059 | 0.0717 | 0.0353 | 0.0211 | 0.0434 | 0.0221 | 0.0401 |
| 6 | 0.0025 | 0.0033 | 0.0044 | 0.0300 | 0.0093 | 0.0016 | 0.0171 | 0.0020 | 0.0144 |
| 7 | 0.0000 | 0.0002 | 0.0219 | 0.0249 | 0.0006 | 0.0001 | 0.0005 | 0.0001 | 0.0010 |
| 8 | 0.0010 | 0.0071 | 0.0240 | 0.0760 | 0.0223 | 0.0151 | 0.0279 | 0.0157 | 0.0154 |
| 9 | 0.0047 | 0.0207 | 0.0150 | 0.0471 | 0.0832 | 0.0262 | 0.1047 | 0.0278 | 0.0721 |
| 10 | 0.0038 | 0.0150 | 0.0206 | 0.0223 | 0.0685 | 0.0180 | 0.0835 | 0.0207 | 0.1278 |
| **ID** | **C′051** | **C′052** | **C′053** | **C′061** | **C′062** | **C′071** | **C′072** | **C′073** | **C′074** |
| 1 | 0.0000 | 0.2308 | 0.0000 | 0.2500 | 0.1000 | 0.0000 | 0.0000 | 0.0000 | 0.0000 |
| 2 | 0.0000 | 0.0000 | 0.0000 | 0.0000 | 0.0000 | 0.0000 | 0.0000 | 0.0000 | 0.0000 |
| 3 | 0.0588 | 0.0385 | 0.0000 | 0.0000 | 0.1000 | 0.0253 | 0.0000 | 0.0000 | 0.0000 |
| 4 | 0.0588 | 0.0000 | 0.0000 | 0.0000 | 0.0000 | 0.0000 | 0.0000 | 0.0000 | 0.0000 |
| 5 | 0.1471 | 0.1154 | 0.0000 | 0.0000 | 0.1000 | 0.0000 | 0.0000 | 0.0253 | 0.0000 |
| 6 | 0.0000 | 0.0385 | 0.0000 | 0.0000 | 0.0000 | 0.0000 | 0.0000 | 0.0000 | 0.0000 |
| 7 | 0.0294 | 0.0000 | 0.0000 | 0.0000 | 0.0000 | 0.0000 | 0.0000 | 0.0000 | 0.0000 |
| 8 | 0.0000 | 0.0000 | 0.0000 | 0.0000 | 0.0000 | 0.0000 | 0.0000 | 0.0000 | 0.0000 |
| 9 | 0.0294 | 0.0769 | 0.0000 | 0.0000 | 0.1000 | 0.0000 | 0.0000 | 0.0000 | 0.0000 |
| 10 | 0.0588 | 0.0000 | 0.0000 | 0.0000 | 0.0000 | 0.0000 | 0.0253 | 0.1610 | 0.0000 |
| **ID** | **C′075** | **C′076** | **C′081** | **C′082** | **C′083** | **C′091** | **C′092** | **C′093** | **C′101** |
| 1 | 0.0000 | 0.2000 | 1.0000 | 0.0000 | 0.0000 | 0.0000 | 0.0000 | 0.0000 | 0.0000 |
| 2 | 0.0000 | 0.0000 | 0.5000 | 0.0000 | 0.0000 | 0.0000 | 0.0000 | 0.0000 | 0.0000 |
| 3 | 0.0000 | 0.2462 | 1.0000 | 0.0000 | 0.1111 | 0.0000 | 0.0000 | 0.0000 | 0.0000 |
| 4 | 0.0000 | 0.0000 | 0.5000 | 0.0000 | 0.1111 | 0.0000 | 0.0000 | 0.0000 | 0.0000 |
| 5 | 0.0000 | 0.0923 | 0.5000 | 0.0000 | 0.0000 | 0.0000 | 0.0000 | 0.0000 | 0.0000 |
| 6 | 0.0000 | 0.1231 | 0.5000 | 0.0000 | 0.0000 | 0.0000 | 0.0000 | 0.0000 | 0.0000 |
| 7 | 0.0000 | 0.0308 | 0.5000 | 0.0000 | 0.1111 | 0.0000 | 0.0000 | 0.0000 | 0.0000 |
| 8 | 0.0000 | 0.0769 | 0.5000 | 0.0000 | 0.3333 | 0.0000 | 0.0000 | 0.0000 | 0.0000 |
| 9 | 0.0000 | 0.4462 | 0.5000 | 0.0000 | 0.0000 | 0.0000 | 0.0000 | 0.0000 | 0.0000 |
| 10 | 0.0000 | 0.0154 | 0.5000 | 0.0000 | 0.3333 | 0.0000 | 0.0000 | 0.0000 | 0.0000 |
| **ID** | **C′111** | **C′112** | **C′113** | **C′121** | **C′122** | **C′131** | **C′132** | **C′133** | **C′134** |
| 1 | 0.0653 | 0.4521 | 0.0027 | 0.6457 | 0.8565 | 0.1215 | 0.1253 | 0.0213 | 0.1025 |
| 2 | 0.0092 | 0.3561 | 0.0000 | 0.2632 | 0.7569 | 0.3645 | 0.8625 | 0.0154 | 0.0125 |
| 3 | 0.0467 | 0.3775 | 0.0915 | 0.5799 | 0.7752 | 0.1021 | 0.2125 | 0.0125 | 0.2153 |
| 4 | 0.0122 | 0.3522 | 0.0012 | 0.3361 | 0.7562 | 0.0463 | 0.1523 | 0.0256 | 0.1025 |
| 5 | 0.0229 | 0.3847 | 0.0093 | 0.7314 | 0.7856 | 0.0153 | 0.1033 | 0.2153 | 0.3256 |
| 6 | 0.0173 | 0.4107 | 0.0166 | 0.6011 | 0.8125 | 0.0103 | 0.1574 | 0.2533 | 0.1253 |
| 7 | 0.0724 | 0.5651 | 0.0008 | 0.6204 | 0.5625 | 0.0562 | 0.215 | 0.1425 | 0.0156 |
| 8 | 0.0979 | 0.4851 | 0.0081 | 0.6420 | 0.8569 | 0.1025 | 0.2152 | 0.3652 | 0.0512 |
| 9 | 0.0609 | 0.2418 | 0.0228 | 0.7016 | 0.6584 | 0.0356 | 0.1361 | 0.2563 | 0.0652 |
| 10 | 0.0535 | 0.3586 | 0.0270 | 0.2499 | 0.7589 | 0.0458 | 0.0586 | 0.1452 | 0.0563 |
| **ID** | **C′135** | **C′136** | **C′141** | **C′142** | **C′143** | **C′151** | **C′161** | **C′171** | **C′181** |
| 1 | 0.0955 | 0.0460 | 0.0040 | 0.0040 | 0.0040 | 0.0000 | 0.0956 | 0.0000 | 0.0943 |
| 2 | 0.4464 | 0.2322 | 0.0025 | 0.0025 | 0.0025 | 0.0000 | 0.1962 | 0.0007 | 0.4208 |
| 3 | 0.2633 | 0.1884 | 0.0004 | 0.0004 | 0.0004 | 1.0000 | 0.1232 | 0.0001 | 0.1827 |
| 4 | 0.0415 | 0.0430 | 0.0006 | 0.0006 | 0.0006 | 0.0000 | 0.0704 | 0.0001 | 0.0241 |
| 5 | 0.0090 | 0.0178 | 0.0071 | 0.0071 | 0.0071 | 0.0000 | 0.0304 | 0.0010 | 0.0163 |
| 6 | 0.0156 | 0.0267 | 0.0000 | 0.0000 | 0.0000 | 0.0000 | 0.0641 | 0.0001 | 0.0136 |
| 7 | 0.0574 | 0.0282 | 0.0017 | 0.0017 | 0.0017 | 0.0000 | 0.2172 | 0.0208 | 0.0666 |
| 8 | 0.1129 | 0.0809 | 0.0024 | 0.0024 | 0.0024 | 0.0000 | 0.0945 | 0.0001 | 0.1043 |
| 9 | 0.0804 | 0.0623 | 0.0019 | 0.0019 | 0.0018 | 0.0000 | 0.1537 | 0.0001 | 0.0694 |
| 10 | 0.0046 | 0.0223 | 0.0011 | 0.0011 | 0.0011 | 0.0000 | 0.0237 | 0.0000 | 0.0088 |

Step 3, measure the proportion of indicators. Calculate the specific gravity Pij of each indicator in the corresponding block, and the calculation formula is as follows:

$P_{\text{ij}}=\frac{X_{\text{ij}}^{'}}{\sum_{1}^{n} X_{\text{ij}}^{'}}$（i=1，2...，n； j=1，2，...，m）	（17）

Where Pij represents the proportion of block i in the index of item j. Table 6 is a detailed presentation of the measurement results of the proportion of indicators.

### Table 6 Detailed presentation of the measurement results of the proportion of selected sample indicators**^[2]^**

| **ID** | **P011** | **P012** | **P13** | **P014** | **P015** | **P021** | **P031** | **P041** | **P042** |
| --- | --- | --- | --- | --- | --- | --- | --- | --- | --- |
| 1 | 0.0000 | 0.0000 | 0.0000 | 0.0000 | 0.0000 | 0.0000 | 0.0000 | 0.0000 | 0.0000 |
| 2 | 0.0000 | 0.0000 | 0.0000 | 0.0000 | 0.0000 | 0.0002 | 0.0001 | 0.0002 | 0.0002 |
| 3 | 0.0000 | 0.0000 | 0.0006 | 0.0001 | 0.0000 | 0.0000 | 0.0001 | 0.0000 | 0.0002 |
| 4 | 0.0002 | 0.0002 | 0.0002 | 0.0001 | 0.0000 | 0.0001 | 0.0002 | 0.0002 | 0.0001 |
| 5 | 0.0001 | 0.0001 | 0.0000 | 0.0000 | 0.0000 | 0.0000 | 0.0001 | 0.0000 | 0.0001 |
| 6 | 0.0000 | 0.0000 | 0.0000 | 0.0000 | 0.0000 | 0.0002 | 0.0001 | 0.0000 | 0.0000 |
| 7 | 0.0000 | 0.0000 | 0.0000 | 0.0000 | 0.0000 | 0.0003 | 0.0002 | 0.0001 | 0.0001 |
| 8 | 0.0000 | 0.0000 | 0.0001 | 0.0001 | 0.0000 | 0.0002 | 0.0001 | 0.0002 | 0.0003 |
| 9 | 0.0000 | 0.0000 | 0.0001 | 0.0000 | 0.0000 | 0.0002 | 0.0001 | 0.0002 | 0.0003 |
| 10 | 0.0000 | 0.0000 | 0.0176 | 0.0030 | 0.0000 | 0.0000 | 0.0002 | 0.0044 | 0.0031 |
| **ID** | **P051** | **P052** | **P053** | **P061** | **P062** | **P071** | **P072** | **P073** | **P074** |
| 1 | 0.0000 | 0.0000 | 0.0000 | 0.0000 | 0.0000 | 0.0000 | 0.0000 | 0.0000 | 0.0000 |
| 2 | 0.0003 | 0.0003 | 0.0023 | 0.0010 | 0.0000 | 0.0000 | 0.0000 | 0.0000 | 0.0000 |
| 3 | 0.0001 | 0.0002 | 0.0023 | 0.0000 | 0.0000 | 0.0000 | 0.0000 | 0.0000 | 0.0000 |
| 4 | 0.0003 | 0.0002 | 0.0057 | 0.0029 | 0.0000 | 0.0000 | 0.0000 | 0.0000 | 0.0000 |
| 5 | 0.0000 | 0.0001 | 0.0000 | 0.0010 | 0.0000 | 0.0000 | 0.0000 | 0.0000 | 0.0000 |
| 6 | 0.0000 | 0.0000 | 0.0011 | 0.0000 | 0.0000 | 0.0000 | 0.0000 | 0.0000 | 0.0000 |
| 7 | 0.0002 | 0.0001 | 0.0000 | 0.0000 | 0.0000 | 0.0000 | 0.0000 | 0.0000 | 0.0000 |
| 8 | 0.0004 | 0.0004 | 0.0011 | 0.0019 | 0.0000 | 0.0000 | 0.0000 | 0.0000 | 0.0000 |
| 9 | 0.0003 | 0.0007 | 0.0023 | 0.0000 | 0.0000 | 0.0000 | 0.0000 | 0.0000 | 0.0000 |
| 10 | 0.0078 | 0.0034 | 0.0000 | 0.0048 | 0.0000 | 0.0000 | 0.0000 | 0.0000 | 0.0000 |
| **ID** | **P075** | **P076** | **P081** | **P082** | **P083** | **P091** | **P092** | **P093** | **P101** |
| 1 | 0.0000 | 0.0000 | 0.0000 | 0.0000 | 0.0000 | 0.0000 | 0.0000 | 0.0000 | 0.0000 |
| 2 | 0.0000 | 0.0001 | 0.0012 | 0.0186 | 0.0058 | 0.0000 | 0.0039 | 0.0000 | 0.0000 |
| 3 | 0.0000 | 0.0000 | 0.0000 | 0.0000 | 0.0029 | 0.0000 | 0.0039 | 0.0000 | 0.0000 |
| 4 | 0.0000 | 0.0001 | 0.0012 | 0.0070 | 0.0000 | 0.0000 | 0.0000 | 0.0000 | 0.0000 |
| 5 | 0.0000 | 0.0000 | 0.0000 | 0.0093 | 0.0029 | 0.0000 | 0.0000 | 0.0000 | 0.0000 |
| 6 | 0.0000 | 0.0000 | 0.0000 | 0.0023 | 0.0029 | 0.0000 | 0.0039 | 0.0000 | 0.0000 |
| 7 | 0.0000 | 0.0000 | 0.0000 | 0.0058 | 0.0029 | 0.0000 | 0.0117 | 0.0000 | 0.0000 |
| 8 | 0.0000 | 0.0001 | 0.0000 | 0.0337 | 0.0029 | 0.0000 | 0.0000 | 0.0000 | 0.0000 |
| 9 | 0.0000 | 0.0000 | 0.0037 | 0.0012 | 0.0029 | 0.0000 | 0.0117 | 0.0000 | 0.0000 |
| 10 | 0.0000 | 0.0000 | 0.0000 | 0.0604 | 0.0000 | 0.0000 | 0.0000 | 0.0000 | 0.0000 |
| **ID** | **P111** | **P112** | **P113** | **P121** | **P122** | **P131** | **P132** | **P133** | **P134** |
| 1 | 0.0000 | 0.0000 | 0.0001 | 0.0000 | 0.0019 | 0.0000 | 0.0000 | 0.0000 | 0.0000 |
| 2 | 0.0000 | 0.0000 | 0.0003 | 0.0000 | 0.0015 | 0.0000 | 0.0001 | 0.0001 | 0.0000 |
| 3 | 0.0000 | 0.0000 | 0.0001 | 0.0000 | 0.0016 | 0.0000 | 0.0000 | 0.0000 | 0.0000 |
| 4 | 0.0000 | 0.0000 | 0.0002 | 0.0000 | 0.0017 | 0.0000 | 0.0000 | 0.0000 | 0.0000 |
| 5 | 0.0000 | 0.0000 | 0.0001 | 0.0000 | 0.0018 | 0.0000 | 0.0000 | 0.0000 | 0.0000 |
| 6 | 0.0000 | 0.0000 | 0.0005 | 0.0000 | 0.0006 | 0.0000 | 0.0000 | 0.0000 | 0.0000 |
| 7 | 0.0000 | 0.0000 | 0.0007 | 0.0000 | 0.0019 | 0.0000 | 0.0000 | 0.0000 | 0.0000 |
| 8 | 0.0000 | 0.0000 | 0.0004 | 0.0000 | 0.0004 | 0.0000 | 0.0000 | 0.0000 | 0.0000 |
| 9 | 0.0000 | 0.0000 | 0.0004 | 0.0000 | 0.0004 | 0.0001 | 0.0000 | 0.0000 | 0.0000 |
| 10 | 0.0019 | 0.0001 | 0.0001 | 0.0001 | 0.0001 | 0.0002 | 0.0001 | 0.0002 | 0.0001 |
| **ID** | **P135** | **P136** | **P141** | **P142** | **P143** | **P151** | **P161** | **P171** | **P181** |
| 1 | 0.0007 | 0.0013 | 0.0032 | 0.0061 | 0.0000 | 0.0000 | 0.0000 | 0.0000 | 0.0006 |
| 2 | 0.0015 | 0.0004 | 0.0026 | 0.0026 | 0.0000 | 0.0000 | 0.0000 | 0.1081 | 0.0004 |
| 3 | 0.0009 | 0.0002 | 0.0006 | 0.0033 | 0.0000 | 0.0000 | 0.0000 | 0.0000 | 0.0002 |
| 4 | 0.0019 | 0.0001 | 0.0002 | 0.0013 | 0.0001 | 0.0000 | 0.0000 | 0.0000 | 0.0001 |
| 5 | 0.0015 | 0.0001 | 0.0004 | 0.0020 | 0.0000 | 0.0000 | 0.0000 | 0.0000 | 0.0002 |
| 6 | 0.0016 | 0.0002 | 0.0004 | 0.0010 | 0.0000 | 0.0000 | 0.0000 | 0.0000 | 0.0006 |
| 7 | 0.0017 | 0.0003 | 0.0011 | 0.0021 | 0.0000 | 0.0000 | 0.0000 | 0.0000 | 0.0003 |
| 8 | 0.0018 | 0.0002 | 0.0009 | 0.0028 | 0.0000 | 0.0000 | 0.0000 | 0.0000 | 0.0004 |
| 9 | 0.0006 | 0.0001 | 0.0003 | 0.0016 | 0.0000 | 0.0000 | 0.0000 | 0.0000 | 0.0001 |
| 10 | 0.0019 | 0.0006 | 0.0018 | 0.0046 | 0.0000 | 0.0000 | 0.0000 | 0.0000 | 0.0017 |

The fourth step is the measurement of information entropy. "Information entropy" is a concept proposed by C.E. Schannon in 1948, which is used to solve the problem of quantitative measurement of information. The measurement of information entropy can evaluate the degree of influence of each index on the quality of a block and the degree of dispersion of each index. The greater the degree of dispersion of an index, the stronger the influence on the quality of a block. Therefore, the weight of each index can be calculated through the measurement of information entropy, which provides a basis for multi-index evaluation of district quality.Item j information entropy entropy value is expressed as ej, expressed in the following formula:

$\text{e}_{\text{j}}=-\frac{1}{\text{ln（n）}}\sum_{\text{i}=1}^{\text{n}} P_{\text{ij}}\text{ln（}P_{\text{ij}}\text{）}$ （18）

Table7 shows the detailed information entropy measurement results.

### Table 7 Information entropy value table

| **e011** | **e012** | **e13** | **e014** | **e015** | **e021** | **e031** | **e041** | **e042** |
| --- | --- | --- | --- | --- | --- | --- | --- | --- |
| 0.8602 | 0.8702 | 0.9357 | 0.5557 | 0.7535 | 0.5622 | 0.9152 | 0.8326 | 0.0394 |
| **e051** | **e052** | **e053** | **e061** | **e062** | **e071** | **e072** | **e073** | **e074** |
| 0.3314 | 0.3187 | 0.4023 | 0.2513 | 0.2657 | 0.8562 | 0.7523 | 0.6552 | 0.6256 |
| **e075** | **e076** | **e081** | **e082** | **e083** | **e091** | **e092** | **e093** | **e101** |
| 0.7236 | 0.1041 | 0.8633 | 0.2506 | 0.0663 | 0.2675 | 0.2233 | 0.2872 | 0.4537 |
| **e111** | **e112** | **e113** | **e121** | **e122** | **e131** | **e132** | **e133** | **e134** |
| 0.2455 | 0.3255 | 0.1758 | 0.0760 | 0.3544 | 0.7966 | 0.8752 | 0.8458 | 0.7523 |
| **e135** | **e136** | **e141** | **e142** | **e143** | **e151** | **e161** | **e171** | **e181** |
| 0.2736 | 0.2315 | 0.6835 | 0.9103 | 0.7411 | 0.1815 | 0.1135 | 0.1524 | 0.7721 |

Step 5, the difference coefficient is measured. The difference coefficient is negatively correlated with entropy. The larger the difference coefficient of the same index is, the smaller the entropy is. Table 8 is a detailed presentation of the measurement results of the difference coefficient.

$d_{j}=1- e_{j}$	（19）

### Table 8 Information entropy difference coefficient table

| **d011** | **d012** | **d13** | **d014** | **d015** | **d021** | **d031** | **d041** | **d042** |
| --- | --- | --- | --- | --- | --- | --- | --- | --- |
| 0.1398 | 0.1298 | 0.0643 | 0.4443 | 0.2465 | 0.4378 | 0.0848 | 0.1674 | 0.9606 |
| **d051** | **d052** | **d053** | **d061** | **d062** | **d071** | **d072** | **d073** | **d074** |
| 0.6686 | 0.6813 | 0.5977 | 0.7487 | 0.7343 | 0.1438 | 0.2477 | 0.3448 | 0.3744 |
| **d075** | **d076** | **d081** | **d082** | **d083** | **d091** | **d092** | **d093** | **d101** |
| 0.2764 | 0.8959 | 0.1367 | 0.7494 | 0.9337 | 0.7325 | 0.7767 | 0.7128 | 0.5463 |
| **d111** | **d112** | **d113** | **d121** | **d122** | **d131** | **d132** | **d133** | **d134** |
| 0.7545 | 0.6745 | 0.8242 | 0.9240 | 0.6456 | 0.2034 | 0.1248 | 0.1542 | 0.2477 |
| **d135** | **d136** | **d141** | **d142** | **d143** | **d151** | **d161** | **d171** | **d181** |
| 0.7264 | 0.7685 | 0.3165 | 0.0897 | 0.2589 | 0.8185 | 0.8865 | 0.8476 | 0.2279 |

Step 6, index weight calculation:

$\omega_{\text{j}}=\frac{\text{d}_{\text{j}}}{\sum_{\text{j}=1}^{\text{m}} \text{d}_{\text{j}}}$ （20）

Table9 shows the detailed measurement results of indicator weights.

### Table 9 Measurement results of index weights

| **ω011** | **ω012** | **ω13** | **ω014** | **ω015** | **ω021** | **ω031** | **ω041** | **ω042** |
| --- | --- | --- | --- | --- | --- | --- | --- | --- |
| 0.0062 | 0.0058 | 0.0029 | 0.0198 | 0.0110 | 0.0195 | 0.0038 | 0.0075 | 0.0427 |
| **ω051** | **ω052** | **ω053** | **ω061** | **ω062** | **ω071** | **ω072** | **ω073** | **ω074** |
| 0.0298 | 0.0303 | 0.0266 | 0.0333 | 0.0327 | 0.0064 | 0.0110 | 0.0153 | 0.0167 |
| **ω075** | **ω076** | **ω081** | **ω082** | **ω083** | **ω091** | **ω092** | **ω093** | **ω101** |
| 0.0123 | 0.0399 | 0.0061 | 0.0334 | 0.0416 | 0.0326 | 0.0346 | 0.0317 | 0.0243 |
| **ω111** | **ω112** | **ω113** | **ω121** | **ω122** | **ω131** | **ω132** | **ω133** | **ω134** |
| 0.0336 | 0.0300 | 0.0367 | 0.0411 | 0.0287 | 0.0091 | 0.0056 | 0.0069 | 0.0110 |
| **ω135** | **ω136** | **ω141** | **ω142** | **ω143** | **ω151** | **ω161** | **ω171** | **ω181** |
| 0.0323 | 0.0342 | 0.0141 | 0.0040 | 0.0115 | 0.0364 | 0.0395 | 0.0377 | 0.0101 |

Step 7, weighted sum, calculate the neighborhood quality index of each block

$JQ_{\text{i}}=\sum_{\text{j}=1}^{\text{m}} \omega_{\text{j}}\times\text{P}_{\text{ij}}$ （21）

Table 10 is a summary of the weight measurement results of neighborhood quality indicators.

### Table 10 Summary of weight measurement results of neighborhood quality indicators

| **Weight of level-1 indicator** | **Weight of level-2 indicator** | **Weight of level-3 indicator** | |
| --- | --- | --- | --- |
| (B1)  Built Environment (0.1191) | C01  Traffic fluency  (0.0456) | C011 Proportion of sidewalks | 0.0062 |
|  |  | C012 Proportion of driveways | 0.0058 |
|  |  | C013 Sidewalk fluency | 0.0029 |
|  |  | C014 Driveway fluency | 0.0198 |
|  |  | C015 Completeness of transportation infrastructure | 0.0110 |
|  | C02 Density of buildings (0.0195) | C021 Proportion of buildings | 0.0195 |
|  | C03 Green visible index (0.0038) | C031 Proportion of plants | 0.0038 |
|  | C04 Permeability of streetscape (0.0502) | C041 Proportion of the sky | 0.0075 |
|  |  | C042 Proportion of street walls | 0.0427 |
| (B2)  Cultural atmosphere  (0.2543) | C05  Cultural facilities  (0.0867) | C051 Museums | 0.0298 |
|  |  | C052 Theaters and cinemas | 0.0303 |
|  |  | C053 Public libraries | 0.0266 |
|  | C06 Historical profundity  (0.0660) | C061 Organizations with modern cultural characteristics | 0.0333 |
|  |  | C062 Organizations under historical and cultural protection | 0.0327 |
|  | C07 Natural resource  (0.1016) | C071 National AAAAA Class Scenic Spot | 0.0064 |
|  |  | C072 National AAAA Class Scenic Spot | 0.0110 |
|  |  | C073 National AAA Class Scenic Spot | 0.0153 |
|  |  | C074 National AA Class Scenic Spot | 0.0167 |
|  |  | C075National A Class Scenic Spot | 0.0123 |
|  |  | C076 Greenland parks | 0.0399 |
| (B3)  Creativity  (0.2042) | C08  University  (0.0810) | C081 985-project universities | 0.0061 |
|  |  | C082 211-project universities | 0.0334 |
|  |  | C083 Other colleges and universities | 0.0416 |
|  | C09  National key laboratory  (0.0989) | C091 State Key Laboratories of enterprises | 0.0326 |
|  |  | C092 State Key Laboratories jointly built by provinces and ministries | 0.0346 |
|  |  | C093 State Key Laboratories in colleges and universities | 0.0317 |
|  | C10 Scientific research institutions  (0.00243) | C101 Institutes of Chinese Academy of Sciences | 0.0243 |
| (B4)  Inclusiveness  (0.2691) | C11  Fairness and justice (0.1003) | C111 Human-vehicle balance | 0.0336 |
|  |  | C112 Proportion of the disadvantaged groups population | 0.0300 |
|  |  | C113 Transportation hubs | 0.0367 |
|  | C12 Respect for diversity (0.00699) | C121 Functional diversity | 0.0411 |
|  |  | C122 Proportion of the outlanders' population | 0.0287 |
|  | C13  People oriented (0.0990) | C131 Level III hospitals | 0.0091 |
|  |  | C132 Level II hospitals | 0.0056 |
|  |  | C133 Level I hospitals | 0.0069 |
|  |  | C134 Other hospitals | 0.0110 |
|  |  | C135 Living services | 0.0323 |
|  |  | C136 Science, education and cultural services | 0.0342 |
| (B5)  Brand image  (0.0660) | C14  Breadth for brand image  (0.00296) | C141 Related searches on Baidu platform in 10 years | 0.0141 |
|  |  | C142 Related searches on Tencent platform in 10 years | 0.0040 |
|  |  | C143 Related searches on 360.cn in 10 years | 0.0115 |
|  | C15 Depth for brand image (0.0364) | C151 National-level awards | 0.0364 |
| (B6)  Business environment  (0.0873) | C16 Commercial vitality (0.395） | C161 Economic development level | 0.0395 |
|  | C17 Business diversity (0.0377) | C171 Business diversity | 0.0377 |
|  | C18 Market size (0.0101) | C181 Number of merchants | 0.0101 |

# Appendix 4：

#### Table 11. Classification of Core Cities and Ordinary Cities in China

| Administrative level | | Cities covered |
| --- | --- | --- |
| Core cities | Municipalities （4） | Beijing、Tianjin、Shanghai、Chongqing |
|  | Vice – provincial（ capital cities）（10） | Shenyang、Changchun、Harbin、Nanjing、Hangzhou、Jinan、Wuhan、Xi’an、Guangzhou、Chengdu |
|  | Vice – provincial(separate-planning cities)（5） | Dalian, Qingdao, Ningbo, Xiamen, Shenzhen |
|  | Ordinary capital cities（17） | Shijiazhuang, Taiyuan, Hohhot, Hefei, Fuzhou, Nanchang, Zhengzhou, Changsha, Nanning, Haikou, Guiyang, Kunming, Lhasa, Lanzhou, Xining, Yinchuan, Urumqi |
| Ordinary cities | Prefecture level cities（301） | Other prefecture-level cities, unions, autonomous prefecture and districts |

Source: China Urban Statistical Yearbook

#### Table 13 Summary of service cities in China

| **Ranking in the country** | **Cities** | **Tertiary Industry in GDP (%)** |
| --- | --- | --- |
|  |  |  |
|  |  |  |
| 1 | Beijing | 83.87 |
| 2 | Haikou | 80.48 |
| 3 | Shanghai | 73.14 |
| 4 | Guangzhou | 72.51 |
| 5 | Sanya | 72.37 |
| 6 | Urumqi | 71.99 |
| 7 | Zhangjiajie | 69.66 |
| 8 | Hangzhou | 68.04 |
| 9 | Hohhot | 66.35 |
| 10 | Harbin | 66.04 |
| 11 | Nanning | 65.76 |
| 12 | Chengdu | 65.72 |
| 13 | Lanzhou | 65.67 |
| 14 | Xining | 65.34 |
| 15 | Tianjin | 64.4 |
| 16 | Kunming | 64.13 |
| 17 | Dingxi | 64.12 |
| 18 | Xi’an | 63.67 |
| 19 | Taiyuan | 63.01 |
| 22 | Shijiazhuang | 62.2 |
| 23 | Shenzhen | 62.13 |
| 24 | Wuhan | 61.84 |
| 25 | Baishan | 61.82 |
| 26 | Jinan | 61.62 |
| 27 | Qingdao | 61.39 |
| 28 | Hefei | 61.06 |
| 29 | Kizilsu Kyrgyz autonomous prefecture | 60.55 |
| 30 | Xiamen | 60.08 |
| 31 | Guyuan | 60.05 |
| 32 | Guiyang | 59.85 |
| 33 | Enshi Tujia and Miao autonomous prefecture | 59.32 |
| 34 | Zhengzhou | 59.04 |
| 35 | longnan | 58.96 |
| 36 | Jinhua | 58.1 |
| 37 | Dehongdai and Jingpo autonomous prefecture | 57.96 |
| 38 | Datong | 57.86 |
| 39 | Qiandongnan Miao and Dong autonomous prefecture | 57.76 |
| 40 | Changsha | 57.48 |
| 41 | Lishui | 57.16 |
| 42 | Tongchuan | 56.98 |
| 43 | Tianshui | 56.83 |
| 44 | Aba autonomous prefecture | 56.64 |
| 45 | Xiangxi Tujia and Miao autonomous prefecture | 56.43 |
| 46 | Wenzhou | 56.42 |
| 47 | Zhangjiakou | 56.34 |
| 48 | Fuzhou | 56.07 |
| 49 | Dandong | 56.05 |
| 50 | Huaihua | 55.83 |
| 51 | Jinzhou | 55.54 |
| 52 | Linzhi | 54.97 |
| 53 | Panjin | 54.88 |
| 54 | Zhuhai | 54.88 |
| 55 | Baotou | 54.87 |
| 56 | Mudanjiang | 54.84 |
| 57 | Kandze Tibetan autonomous prefecture | 54.7 |
| 58 | Guilin | 54.44 |
| 59 | Hengyang | 54.36 |
| 61 | Quzhou | 54.14 |
| 62 | Zhangye | 53.84 |
| 63 | Yinchuan | 53.77 |
| 64 | Heyuan | 53.6 |
| 65 | Hengshui | 53.56 |
| 66 | Yangquan | 53.54 |
| 67 | Qinhuangdao | 53.46 |
| 68 | Dalian | 53.43 |
| 69 | Jieyang | 53.42 |
| 70 | Tongren | 53.15 |
| 71 | Wuwei | 53.15 |
| 72 | Anshan | 53.02 |
| 73 | Chongqing | 52.82 |
| 74 | Cangzhou | 52.74 |
| 75 | Lijiang | 52.58 |
| 76 | Danzhou | 52.53 |
| 77 | Suzhou | 52.5 |
| 78 | Wuxi | 52.48 |
| 79 | Pingliang | 52.44 |
| 80 | Haibei Tibetan autonomous prefecture | 52.16 |
| 81 | Yulin | 51.87 |
| 82 | Yantai | 51.83 |
| 83 | Weifang | 51.57 |
| 84 | Changzhou | 51.57 |
| 85 | Weihai | 51.49 |
| 86 | Anshun | 51.48 |
| 87 | Ningbo | 51.39 |
| 88 | Liaocheng | 51.27 |
| 89 | Nanyang | 51.26 |
| 90 | Shaoyang | 51.2 |
| 91 | Shaoxing | 51.16 |
| 92 | Huaibei | 51.09 |
| 93 | Shaoguan | 51.02 |
| 94 | Jilin | 50.96 |
| 95 | Bazhong | 50.88 |
| 96 | Zhoushan | 50.86 |
| 97 | Bozhou | 50.84 |
| 98 | Ziyang | 50.79 |
| 99 | Taizhou | 50.73 |
| 100 | Shangrao | 50.04 |

# Appendix 5

#### Table 14 Comparison of quality scores of high- and low-quality neighborhoods in China

| **Object** | **Score Percentage of Built Environment** | **Score Percentage of Cultural Atmosphere** | **Score Percentage of Creativity** | **Score Percentage of Inclusiveness** | **Score Percentage of Brand Image** | **Score Percentage of Business Environment** |
| --- | --- | --- | --- | --- | --- | --- |
| Top 500 | 16.73% | 16.01% | 16.85% | 26.21% | 9.01% | 15.19% |
| Bottom 500 | 18.48% | 11.96% | 2.31% | 51.35% | 5.82% | 10.08% |

#### Table 15 Comparison of quality scores of neighborhoods in eastern, central, and western China

| **Object** | **Score Percentage of Built Environment** | **Score Percentage of Cultural Atmosphere** | **Score Percentage of Creativity** | **Score Percentage of Inclusiveness** | **Score Percentage of Brand Image** | **Score Percentage of Business Environment** |
| --- | --- | --- | --- | --- | --- | --- |
| Eastern region | 17.72% | 14.86% | 19.32% | 21.71% | 10.07% | 16.32% |
| Central region | 16.82% | 22.23% | 10.04% | 24.82% | 11.91% | 14.18% |
| Western region | 15.77% | 10.68% | 5.12% | 50.01% | 6.38% | 12.04% |

Table 16 Comparison of Neighborhood Quality Scores for Heterogeneous Neighborhoods in East, Central, and West Regions

| **Object** | | **Score Percentage of Built Environment** | **Score Percentage of Cultural Atmosphere** | **Score Percentage of Creativity** | **Score Percentage of Inclusiveness** | **Score Percentage of Brand Image** | **Score Percentage of Business Environment** |
| --- | --- | --- | --- | --- | --- | --- | --- |
| Eastern regions | Busy districts | 15.23% | 15.12% | 21.11% | 19.25% | 11.42% | 17.87% |
|  | Developing districts | 18.75% | 13.25% | 19.52% | 21.45% | 10.25% | 16.78% |
|  | Underdeveloped districts | 15.42% | 8.54% | 15.42% | 38.56% | 8.54% | 13.52% |
| Central regions | Busy districts | 17.56% | 23.65% | 11.52% | 19.68% | 12.36% | 15.23% |
|  | Developing districts | 16.98% | 20.12% | 10.25% | 30.44% | 9.56% | 12.65% |
|  | Underdeveloped districts | 13.52% | 16.23% | 8.45% | 45.26% | 5.45% | 11.09% |
| Western regions | Busy districts | 18.97% | 12.35% | 13.54% | 32.15% | 8.56% | 14.43% |
|  | Developing districts | 23.54% | 10.22% | 4.23% | 45.86% | 5.12% | 11.03% |
|  | Underdeveloped districts | 12.54% | 9.54% | 2.54% | 62.35% | 2.52% | 10.51% |

# Appendix 6

**Step 1**: data collection and organization. Matrix arrangement is made for all the data. $X_{\mathrm{ij}}$in the matrix represents the value of the j-th indicator in the i-th neighborhood (i=1, 2... , n; j=1, 2, ... , m).

$\text{A}=\left(\begin{matrix} \text{X}_{11} & ... & \text{X}_{\text{1m}} \\ ... & ... & ... \\ \text{X}_{\text{n1}} & ... & \text{X}_{\text{nm}} \end{matrix}\right)_{\text{n}\times\text{m}}$ （1）

**Step 2:** standardization of basic data. The specific algorithm is as follows:

$X_{ij}^{’}=\frac{X_{ij}-min（X_{1j}，...X_{nj}）}{max（X_{1j}，...X_{nj}）-min（X_{1j}，...X_{nj}）}$	（2）

$X_{\mathrm{ij}}^{’}$ is the normalized data. $A^{'}$ is the normalized data matrix, with the following expression:

$A^{'}=\left(\begin{matrix} X^{'}_{11} & ... & X^{'}_{1m} \\ ... & ... & ... \\ X^{'}_{n1} & ... & X^{'}_{nm} \end{matrix}\right)_{\text{n}\times\text{m}}$ （3）

**Step 3:** calculation of indicator proportion. $P_{\mathrm{ij}}$, the proportion of each indicator in the related neighborhood, is calculated in the following formula:

$P_{\text{ij}}=\frac{X_{\text{ij}}^{'}}{\sum_{1}^{n} X_{\text{ij}}^{'}}$（i=1，2...，n； j=1，2，...，m）	（4）

Where $P_{\mathrm{ij}}$ denotes the proportion of the i-th neighborhood's j-th indicator in the total sum of the j-th indicator.

**Step 4**: calculation of information entropy. Through the measurement of information entropy, the weight of each indicator can be calculated, so as to provide a basis for the multi-indicator evaluation of neighborhood quality.The information entropy value of the j-th indicator is expressed as $e_{j}$, and the expression formula is as follows:

$\text{e}_{\text{j}}=-\frac{1}{\text{ln（n）}}\sum_{\text{i}=1}^{\text{n}} P_{\text{ij}}\text{ln（}P_{\text{ij}}\text{）}$ （5）

**Step 5:** calculation of variation coefficient. This coefficient is negatively correlated with the entropy value. The larger the variation coefficient of the same indicator, the smaller the entropy value.

$d_{j}=1- e_{j}$	（6）

**Step 6**: calculation of index weights.

$\omega_{\text{j}}=\frac{\text{d}_{\text{j}}}{\sum_{\text{j}=1}^{\text{m}} \text{d}_{\text{j}}}$ （7）

**Step 7:** weighted summation. The neighborhood quality index of each neighborhood is calculated:

$JQ_{\text{i}}=\sum_{\text{j}=1}^{\text{m}} \omega_{\text{j}}\times\text{P}_{\text{ij}}$ （8）

$\mathrm{JQ}_{i}$ denotes the comprehensive quality score of neighborhood i. The larger the value of the score, the higher the quality of the neighborhood.

# Appendix 7

To further examine the relationship between neighborhood quality and population density in lower-ranked neighborhoods, we select the 500 neighborhoods with the lowest quality scores as a subsample and estimate a regression model to explore their statistical association. The model is specified as follows:

$\text{Q}_{\text{i}}\text{=}\text{α}\text{+}\alpha_{\text{1}}\text{ln}\text{\_}\text{Pop}_{i}\text{+}\text{α}_{\text{2}}\text{ln}\text{\_}\text{GDP}_{\text{c}}\text{+}\text{α}_{\text{3}}\text{TertiaryS}\text{h}\text{ar}\text{e}_{\text{c}}\text{+}\text{α}_{\text{4}}\text{CoreCit}\text{y}_{\text{c}}\text{+}\text{α}_{\text{5}}\text{Nig}\text{h}\text{tLig}\text{h}\text{t}_{\text{c}}\text{+}\text{α}\text{6}\text{Regio}\text{n}_{\text{c}}\text{+}\mu_{\text{i}}$ （9）

where$\text{Q}_{\text{i}}$denotes the neighborhood quality score for neighborhood i. capturing the level of economic development. $\text{ln\_}\text{Pop}_{i}$denotes the logarithm of the resident population in neighborhood i, c is the city where the block i is located.$\text{ln\_}\text{GDP}_{\text{c}}$ represents the logarithm of GDP per capita in city c, reflecting population agglomeration. $\text{TertiaryShar}\text{e}_{\text{c}}$ refers to the share of the tertiary industry in city c, measuring the structure of the local economy. $\text{CoreCit}\text{y}_{\text{c}}$ is a dummy variable indicating city administrative level, which takes the value of 1 if the city is classified as a core city and 0 otherwise. $\text{NightLigh}\text{t}_{\text{c}}$ represents nighttime light intensity in city c, serving as a proxy for local economic activity and development intensity. $\text{Regio}\text{n}_{\text{c}}$ is a regional dummy variable, which equals 1 if the city is located in eastern China and 0 otherwise. $\text{e}_{\text{i}}$ is the error term.All regressions are estimated using ordinary least squares (OLS), with standard errors clustered at the city level.

Table 17 Subsample regression results for lower-ranked neighborhoods

|  | (1) |
| --- | --- |
|  | modelmain1 |
| VARIABLES | Q |
|  |  |
| ln(Popi) | -0.000510** |
|  | (0.000247) |
| ln(GDPc) | 0.000579* |
|  | (0.000300) |
| TertiaryShare | 7.40e-05*** |
|  | (2.01e-05) |
| CoreCity | 0.000555* |
|  | (0.000284) |
| NightLighti | 9.28e-06 |
|  | (1.78e-05) |
| Region | 3.99e-05 |
|  | (0.000163) |
| Constant | 0.0103*** |
|  | (0.00212) |
| City | Yes |
| Observations | 500 |
| R-squared | 0.804 |

Standard errors in parentheses *** p<0.01, ** p<0.05, * p<0.1

Table 17 reports the regression results based on the subsample of the 500 lowest-ranked neighborhoods.The results show that population density is negatively and significantly associated with neighborhood quality within this subsample. This suggests that, among lower-ranked neighborhoods, areas with higher population concentration tend to exhibit relatively lower quality scores. This finding provides supporting evidence for the interpretation proposed in the previous section, namely that relatively lower population density may be associated with more balanced access to basic neighborhood resources in such contexts.GDP per capita remains positively and weakly significant, indicating that even within lower-ranked neighborhoods, economic development continues to play a role in shaping neighborhood quality. The share of tertiary industry is positively and strongly significant, suggesting that service-oriented economic structures contribute to improving neighborhood conditions, even in relatively disadvantaged areas.Core cities also exhibit higher neighborhood quality within the subsample, reflecting their continued advantages in resource allocation and public service provision. In contrast, nighttime light intensity and regional variables are not statistically significant, suggesting that their effects may be less pronounced within lower-ranked neighborhoods.Overall, these results provide additional evidence on the relationship between population density and neighborhood quality in lower-performing areas. However, it should be noted that these findings reflect statistical associations rather than causal relationships.

# References

Cao,G.,& Zhuo, J.(2017). Inclusive Urban Street Planning With Planning. *Careplanner, 33*(09), 16-21. (in Chinese )

Catherine, H.,& Elli, T. (2019). Inclusive Design: Beyond Accessibility. *World Architecture,*(10):20-25.

Chen,L.et al.(2017). Deeplab: Semantic image segmentation with deep convolutional nets, atrous convolution, and fully connected crfs. *IEEE transactions on pattern analysis and machine intelligence, 40*(4), 834-848.

Fensham, P. J. (2011). Globalization of science education: Comment and a commentary. *Journal of Research in Science teaching, 48*(6), 698-709.

Gehl, J. . (1987). Life Between Buildings: Using Public Space. *Island Press.*

Gong,F.et al.(2018). Mapping sky, tree, and building view factors of street canyons in a high-density urban environment. *Building and Environment, 134,* 155-167.

Li, K., Chen, S., & Li X.(2019). Urban Street Quality Indicators and Measurement. *MethodsStatistics and decision-making, 35*(11),56-59. (in Chinese )

Li, X.et al.(2013). Potential of NPP-VIIRS nighttime light imagery for modeling the regional economy of China. *Remote Sensing, 5*(6), 3057-3081.

Li,X.et al.(2015). Assessing street-level urban greenery using Google Street View and a modified green view index. *Urban Forestry & Urban Greening, 14*(3), 675-685.

Liang, J.et al.(2017). Automatic sky view factor estimation from street view photographs—A big data approach. *Remote Sensing, 9*(5), 411.

Liu, L., Silva, E. A., Wu, C., & Wang, H. (2017). A machine learning-based method for the large-scale evaluation of the qualities of the urban environment. *Computers, environment and urban systems, 65,* 113-125.

Long, Y., & Liu, L. (2017). How green are the streets? An analysis for central areas of Chinese cities using Tencent Street View. *PloS one, 12*(2), e0171110.

Montgomery, J. (2003). Cultural quarters as mechanisms for urban regeneration. Part 1: Conceptualising cultural quarters. *Planning, practice & research, 18*(4), 293-306.

Naik, N., Philipoom, J., Raskar, R., & Hidalgo, C. (2014). Streetscore-predicting the perceived safety of one million streetscapes. *In Proceedings of the IEEE conference on computer vision and pattern recognition workshops* (pp. 779-785).

Qi L.et al.(2009). Research on the Measurement of the Impact of a Mega-Event on the Awareness of Tourism Attractions in Host City. *Journal of Peking University (Natural Science Edition), 45*(06),1061-1067. (in Chinese )

Qian L., & He Y,. (2010). Evaluation of the Impact of 5A-level Tourist Attractions on City Awareness. *Economic Rerearch Guide,* (23),119-121. (in Chinese )

Quercia D, Pesce J P, Almeida V, et al. (2013). Psychological Maps 2.0: A web gamification enterprise starting in London*. In Proceedings of ACM Conference on World Wide Web* *(WWW)*. 10: 2488388.2488481.

Richards, D. R., & Edwards, P. J. (2017). Quantifying street tree regulating ecosystem services using Google Street View. *Ecological indicators, 77,* 31-40.

Tang, J., & Long, Y. (2019). Measuring visual quality of street space and its temporal variation: Methodology and its application in the Hutong area in Being. *Landscape and Urban Planning, 191*, 103436.

Wada, B. K., & Venneri, S. L. (1997). Recent progress in adaptive structures for space. *Acta astronautica, 40*(2-8), 85-94.

Xia, R., & He, Y. (2009). On the Quality Characteristics and Connotation of High-Quality Universities. *Journal of Shanghai Normal University (Philosophy and Social Science Edition),38*(04), 117-125. (in Chinese )

Yan, X. Q., Liu, X. G., Li, H. M., & Li, S. (2024). An osm road data quality assessment model based on reference data. *Journal of Information Engineering University* (001), 025.

Ye, Y., Zeng, W., Shen, Q., Zhang, X., & Lu, Y. (2019). The visual quality of streets: A human-centred continuous measurement based on machine learning algorithms and street view images. *Environment and Planning B: Urban Analytics and City Science, 46*(8), 1439-1457.

Zhao, M., & Chen, R. (2023).Theoritical Progress and Planning Prospect of City Image in the Network Society [J].*Humanities and Geography,38*(01), 71-78.(in Chinese )

Zheng, S., Zhang, X., Sun, W., & Lin, C. (2019). Air pollution and elite college graduates’ job location choice: Evidence from China. *The Annals of Regional Science, 63,* 295-316.

1. The data in the table is reserved to 4 decimal places. Some data less than 0.00005 will be displayed as 0.0000.
2. The data in the table is reserved to 4 decimal places. Some data less than 0.00005 will be displayed as 0.0000.
